# Diverse feasting networks at the end of the Bronze Age in Britain (c. 900-500 BCE) evidenced by multi-isotope analysis

**DOI:** 10.1016/j.isci.2025.113271

**Published:** 2025-09-10

**Authors:** Carmen Esposito, Angela L. Lamb, Morten B. Andersen, Marc-Alban Millet, Edward Inglis, Federico Lugli, Alexandra J. Nederbragt, Richard Madgwick

**Affiliations:** 1 School of History, Archaeology and Religion, Cardiff University, Cardiff, UK; 2 Department of Cultural Heritage, University of Bologna, Ravenna, Italy; 3 National Environmental Isotope Facility, British Geological Survey, Keyworth, Nottingham, UK; 4 School of Earth and Environmental Sciences, Cardiff University, Cardiff, UK; 5 Analytical Instruments Group, Thermo Fisher Scientific, Bremen, Germany; 6 Institute of Geosciences, Goethe University Frankfurt, Frankfurt am Main, Germany; 7 Frankfurt Isotope and Element Research Center (FIERCE), Goethe University Frankfurt, Frankfurt am Main, Germany; 8 Department of Chemical and Geological Science, University of Modena and Reggio Emilia, Modena, Italy

**Keywords:** Isotope chemistry, Social sciences, Archeology

## Abstract

During the Bronze Age-Iron Age transition, climatic change and economic upheaval signaled societal shifts across Europe. Longstanding trade networks broke down and in southern Britain new sites, termed middens, emerged. These vast mounds of cultural debris represent the coming together of vast numbers of people and animals for feasts on a scale unparalleled in British prehistory. Faunal remains are key for assessing the catchments of these feasting events and the scale and nature of community connectivity. This study examines networks and scales of mobility that centered on these enigmatic sites through analysis of the largest multi-isotope dataset on faunal remains (*n* = 254) yet generated in archaeology, aided by a random forest ^87^Sr/^86^Sr isoscape of Britain. The data evidence diverse site roles, with some middens anchoring wide-ranging networks and others being local centers for specialist economies, providing nuanced resolution into the social and economic dynamics of this transitional phase.

## Highlights

• Multi-isotope analysis of fauna from middens reveals varied catchments for feasts
• Sites had diverse roles, some as regional hubs, others as specialist producer sites
• Species-specific networks: pigs and cattle having wide catchments at different sites
• New random forest ^87^Sr/^86^Sr isoscape of Britain used for exploring animal origins

## Graphical abstract

**Figure:**
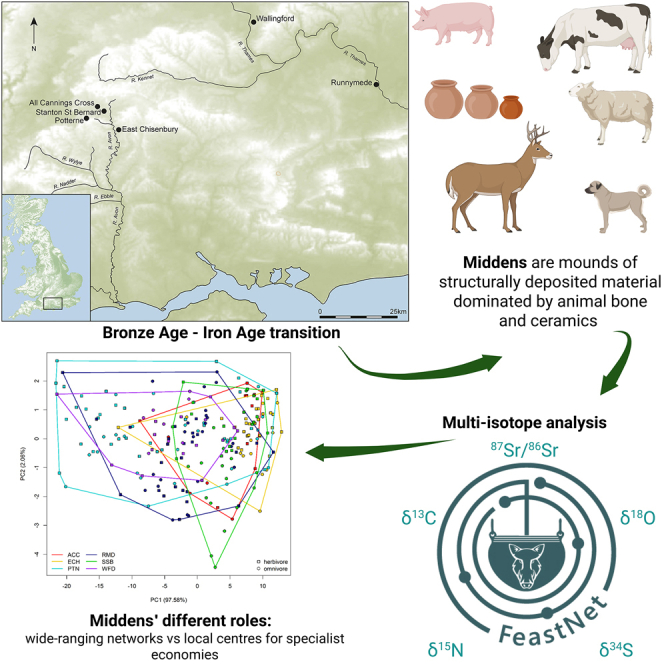

## Introduction

**Figure 1:**
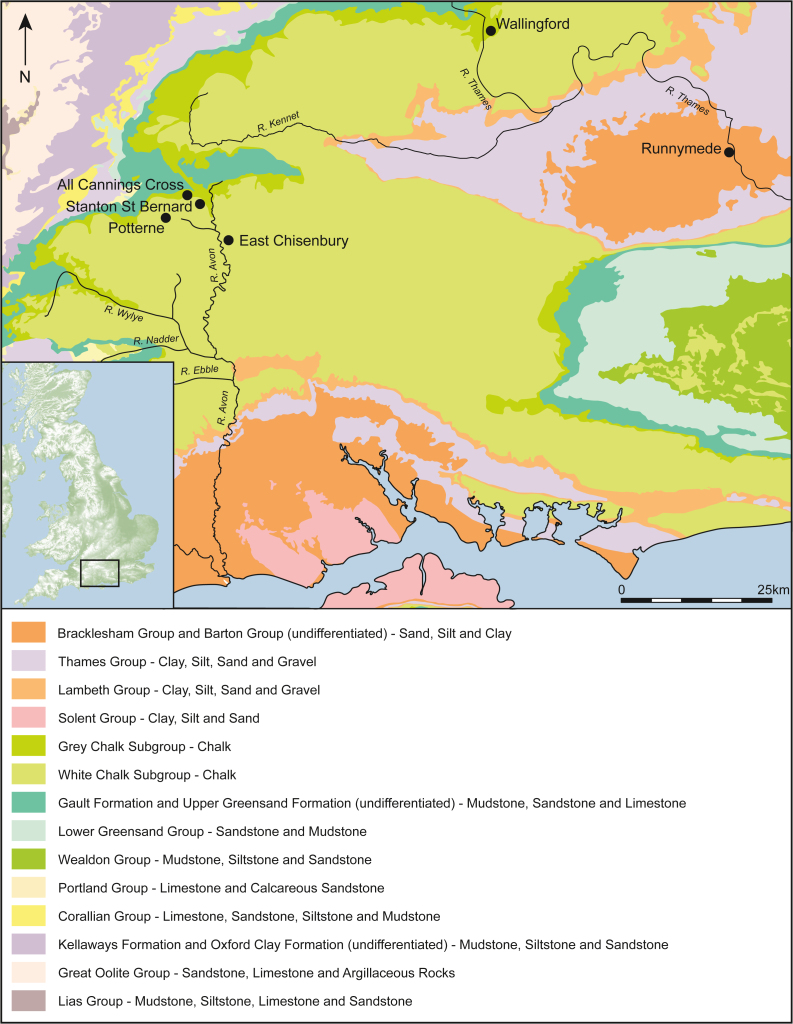
Geological map with analysed midden sites In Wiltshire: All Cannings Cross, East Chisenbury, Potterne, and Stanton St Bernard. In the Thames Valley: Runnymede and Whitecross Farm, Wallingford (British Geological Survey materials © UKRI 2023, inset map contains OS data © Crown copyright and database right [2009], produced by Kirsty Harding).

Understanding the dynamics of the Bronze Age-Iron Age transition has been a longstanding challenge in European archaeology.^1^ The archaeological record shows striking changes in settlement patterns, territorial occupation, and marked regional variation in the character of change.^1^ These shifts are accompanied by rapid climate change,^2^^,^^3^^,^^4^ identifiable around 1550–550 BCE, with 1250–1150 BCE, being a critical transition.^5^^,^^6^ In Britain, multiple datasets show, as a general pattern, a wet period (1500–1200 BCE) followed by a drier one (1200–800 BCE).^5^ In the Late Bronze Age-Early Iron Age transition (LBA-EIA; 900–500 BCE), a significant rapid climate deterioration is evidenced, characterized by a shift to wetter conditions,^4^^,^^7^^,^^8^ which would have had implications for agricultural practices and productivity.^9^ During this period, the Bronze Age regional, island-wide, and continental networks^10^ focused on metalwork decreased, changing in character, while a new site type appeared in southern Britain commonly known as a midden. Middens are vast mounds of structurally deposited material^11^^,^^12^^,^^13^^,^^14^ dominated by significant quantities of animal bone and large quantities of bronze artifacts and ceramics,^15^ hinting at the increasing importance of settlements’ monumentality.^9^ These accumulations of debris point to communal consumption and social mobilization on a very large scale that is arguably unparalleled in British prehistory. Numerous middens have been found in the Vale of Pewsey, in Wiltshire near the landscapes of Stonehenge and Avebury that were the site of vast ritual feasting some 2000 years earlier. The Thames Valley is recognized as another major center of activity (Figure 1). Excavations in these areas have unearthed a great corpus of artifacts and animal bones with several sites producing hundreds of thousands of finds (see^16^ and related bibliography). All these sites represent a comparable phenomenon that highlights the social arena of feasting and symbolic conspicuous consumption,^17^ though there is certainly variation in their character and depositional histories.^12^^,^^16^

Midden sites are representative of a societal shift toward agricultural production, pointing to intensification and a desire to maximize productivity to support ostentatious feasts.^17^ Middens represent the social arena of feasting through significant deposition of animal bones, objects, and artifacts representative of conspicuous consumption.^17^ Zooarchaeological research has provided some information on husbandry regimes (e.g.,^18^^,^^19^^,^^20^^,^^21^), and taphonomic work on faunal remains has helped elucidate the scale, rhythm, and frequency of feasting events.^12^^,^^14^^,^^22^ The continuity of practices and the presence of dark anthropogenic soils are typical traits of middens, as is the close association to rivers; however, the makeup, characteristics, and methods of deposition vary widely both across and within different regions. These sites are often dominated by dark earth and frequently lack clear stratigraphy. Much of the faunal remains derive from relatively homogenous deposits. Taphonomic research suggests large deposits punctuated by hiatuses are characteristic of middens.^12^^,^^14^ This is very much consistent with feasting being the main reason for the accumulations, though it is not possible to confidently assign all faunal remains as feasting debris.

In Wiltshire, East Chisenbury^11^^,^^23^^,^^24^ and Potterne^25^ are two of the largest and most artefact-rich middens in England, whereas All Cannings Cross^26^^,^^27^ and Stanton St Bernard^27^^,^^28^ have more modest assemblages. East Chisenbury is a monumental mound with a diameter of >150 m^11^ and a depth up to 3 m.^24^ Caprines represent the most prevalent taxon, with an unusually large proportion of fetal/neonatal specimens,^11^^,^^23^ suggesting an important sheep dairying element in the economy.^18^^,^^19^ Potterne, located approximately 15 km from East Chisenbury, is another mound of monumental proportions. Numerous animal bones were recovered at the site (c.134,000) dominated by caprines, pigs and cattle.^20^ Pigs are better represented than at most later prehistoric sites in Britain and some were not locally raised.^29^^,^^30^ Unlike East Chisenbury, older sheep/goat, and pigs are common,^20^ suggesting the importance of wool in the economy.^28^ Stanton St. Bernard shows a more substantial ceramic assemblage than the neighboring site of All Cannings Cross. Both ceramics and bones were less abraded than at All Cannings Cross, suggesting a more rapid burial.^31^ The faunal assemblage is dominated by caprines (many of which were juvenile), cattle, and pig.

In the Thames Valley, Runnymede^15^ in the county of Surrey has a wealth of artifacts, while Wallingford^32^ in Oxfordshire is more modest (see^16^ for more detailed information). The site of Runnymede is characterized by a dark earth deposit containing a substantial number of artifacts and animal bones, indicating episodic deposition, as well as structural evidence.^15^^,^^33^^,^^34^ Pigs are found in a higher proportion compared to other late prehistoric Britain sites, but still in smaller numbers compared to caprines and similar numbers to cattle.^35^ Whitecross Farm, Wallingford, is an island settlement with rich ceramic and bone deposits and some metalwork. The LBA faunal assemblage is dominated by caprines and cattle with a notably high proportion of pig remains.^36^ Caprines and pigs younger than two years of age are particularly common.^35^^,^^36^

Scholars have interpreted middens as representing a change in economic focus from previous Bronze Age networks,^7^^,^^37^^,^^38^ pointing to a shift toward agricultural intensification and communal feasting. The nature of midden deposits signals the coming together of vast numbers of people and animals for feasts on a scale unparalleled in British prehistory, arguably even larger than those evidenced in Late Neolithic Britain.^39^ Middens are regarded as focal meeting places to forge relationships and alliances, share and perpetuate knowledge, make artifacts and feast.^9^ An important sense of place was created by seasonal occupation and a long history of accumulation. Middens became landmarks where people met and engaged in acts of conspicuous consumption and display.^9^ Nonetheless, the catchments of these events and the nature of networks underpinning feasting remain poorly understood, since only limited isotope studies have been undertaken and the vast majority of studies focus on carbon (δ^13^C) and nitrogen (δ^15^N) isotopes to explore animal husbandry. As part of the UK Arts and Humanities Research Council (AHRC)-funded FEASTNET (Feasting Networks and Resilience at the end of the British Bronze Age) project, this paper explores animal mobility (*n* = 254) at six midden sites from Wiltshire and the Thames Valley regions through radiogenic strontium (^87^Sr/^86^Sr) and oxygen (δ^18^O) isotope analyses, integrated with sulfur (δ^34^S), carbon and nitrogen (δ^13^C, δ^15^N) isotope analyses of the same animals.^16^ The ^87^Sr/^86^Sr and δ^18^O analyses provide geological and climatic signals for origins,^40^^,^^41^^,^^42^ while δ^34^S shows whether individuals were raised in coastal, wetland or inland areas.^43^^,^^44^ The δ^13^C and δ^15^N also reconstruct the husbandry and foddering regimes for animals at middens and provide an important baseline from which to interpret ^87^Sr/^86^Sr and δ^18^O data.^16^^,^^45^

This research examines the new networks, anchored on these feasting sites that emerged during the LBA-EIA transition in response to climatic and economic change and the catchments from which animals were drawn to be feasted on. The study presents the largest faunal multi-isotope dataset yet generated in archaeology to explore the nature of connectivity at both an intra- and inter-site scale. Species-specific practices of supply are also investigated, as are structured practices relating to management consumption, processing, and deposition of certain taxa that have been established at some middens.^13^^,^^16^^,^^46^ The combination of various proxies, each providing a distinct source of evidence, gives a more nuanced resolution to explore the volume and scale of movement and the type of networks that are anchored on feasting events.^39^^,^^47^ The principal aim of the study is to reconstruct patterns of animal mobility and management across middens during the LBA-EIA transition. This will, in turn, be used to examine the complementary role of different sites, the degree of inter-regional connectivity and what this means for society and economy at this transitional phase. From a methodological perspective, the objective of the study is to assess the potential of a multi-isotope approach combined with biosphere sampling to provide greater resolution on patterns of mobility and move beyond the dichotomy of local versus non-local.

## Results

### Geological and isoscape information for Wiltshire and the Thames Valley

General information on bedrock geology and superficial deposits of the sites is briefly reported for integration with ^87^Sr/^86^Sr, δ^18^O and δ^34^S isoscape information in Table 1. These data are from the BGS geological and isoscape maps (https://www.bgs.ac.uk/datasets/biosphere-isotope-domains-gb/;^48^).

**Table 1:** Summary of the geological and isotope data for the midden sites analyzed in this work

Site	Bedrock geology and superficial deposits description	BGS^87^Sr/^86^Sr	BGS δ^18^O (‰) V-SMOW drinking water zone	BGS δ^34^S (‰) V-CDT	FEASTNET Plants ^87^Sr/^86^Sr	FEASTNET Plants δ^34^S (‰) V-CDT	Combined BGS & FEASTNET ^87^Sr/^86^Sr	Combined BGS & FEASTNET δ^34^S (‰) V-CDT
All Cannings Cross and Stanton St Bernard	ZigZag chalk formation, a sedimentary bedrock formed during the Cretaceous period West Melbury Marly chalk formation of sedimentary origin formed during the Cretaceous period	0.7077 to 0.7094	−7.5 to −7.0	2.4 to 6.4	0.7076 to 0.7078	7.2 to 7.5	0.7076 to 0.7094	2.4 to 7.5
East Chisenbury	Holywell Nodular chalk sedimentary formation formed during the Cretaceous period and superficial Quaternary/Holocene alluvial clay, sand and gravel	0.7077 to 0.7094	−7.5 to −7.0	4.5 to 6.4	0.7078 to 0.7081	4.5 to 6.0	0.7077 to 0.7094	4.5 to 6.4
Potterne	Upper Greensand formation – sandstone, and glauconitic: Sedimentary bedrock formed between during the Cretaceous	0.7078 to 0.7111	−7.5 to −7.0	2.4 to 4.5	0.7087–0.7088	−1.5 to 5.5	0.7078 to 0.7111	−1.5 to 5.5
Runnymede	Kellaways Formation made of sandstone, siltstone and mudstone – sedimentary bedrock formed during the Jurassic period	0.7083 to 0.7101	−7.5 to −7.0	−2.7 to 0.1	0.7089 to 0.7093	−1.0 to 0.1	0.7083 to 0.7101	−2.7 to 0.1
Wallingford	Glauconitic Marl Member – sandstone, glauconitic of sedimentary formation formed during the Cretaceous.	0.7080 to 0.7122	−7.5 to −7.0	−2.7 to 0.1	0.7080 to 0.7085	−3.2 to 5.2	0.7080 to 0.7122	−3.2 to 5.2

Summary of bedrock geology and superficial deposits retrieved from (https://www.bgs.ac.uk/datasets/biosphere-isotope-domains-gb/;^48^). BGS presents ^87^Sr/^86^Sr values of plants. BGS δ^18^O and BGS δ^34^S express values of drinking water and of plants respectively and are reported in ‰ (per mil) (BGS©UKRI). Plants were collected at the midden sites for constructing ^87^Sr/^86^Sr and δ^34^S baselines for each site (c. 5 km radius) and are reported here as FEASTNET plants. BGS and FEASTNET values are combined to define a conservative site local range

#### ^87^Sr/^86^Sr and δ^34^S baseline data: FEASTNET project plants and BGS samples

Full details of the plant samples, geographic coordinates and results are reported in Table S1 with a summary of the limitations of mapping relating to different proxies also provided in the limitations of the study section and the supplementary text. Plant ranges are summarized in Table 1. Plants were collected to define the ^87^Sr/^86^Sr and δ^34^S baseline for each site (c. 5 km radius, Table S1; Figure S1). Sampling focused on site locations and adjacent lithologies. A mixed plant approach was taken to avoid skewed results from outliers and to provide balanced coverage of bioavailability.^49^^,^^50^^,^^51^^,^^52^

The BGS and primary project ^87^Sr/^86^Sr and δ^34^S plant values are combined to define a conservative range for the vicinity of the sites (Table 1). δ^34^S mapping is in its infancy and issues of modern pollution and period-specific δ^34^S cycling mean the degree to which modern baseline plants can be compared to prehistoric animal samples is questionable. They should at least provide an ordinal index of variation. The ^87^Sr/^86^Sr baseline is considered more secure.

#### Faunal results

**Figure 2:**
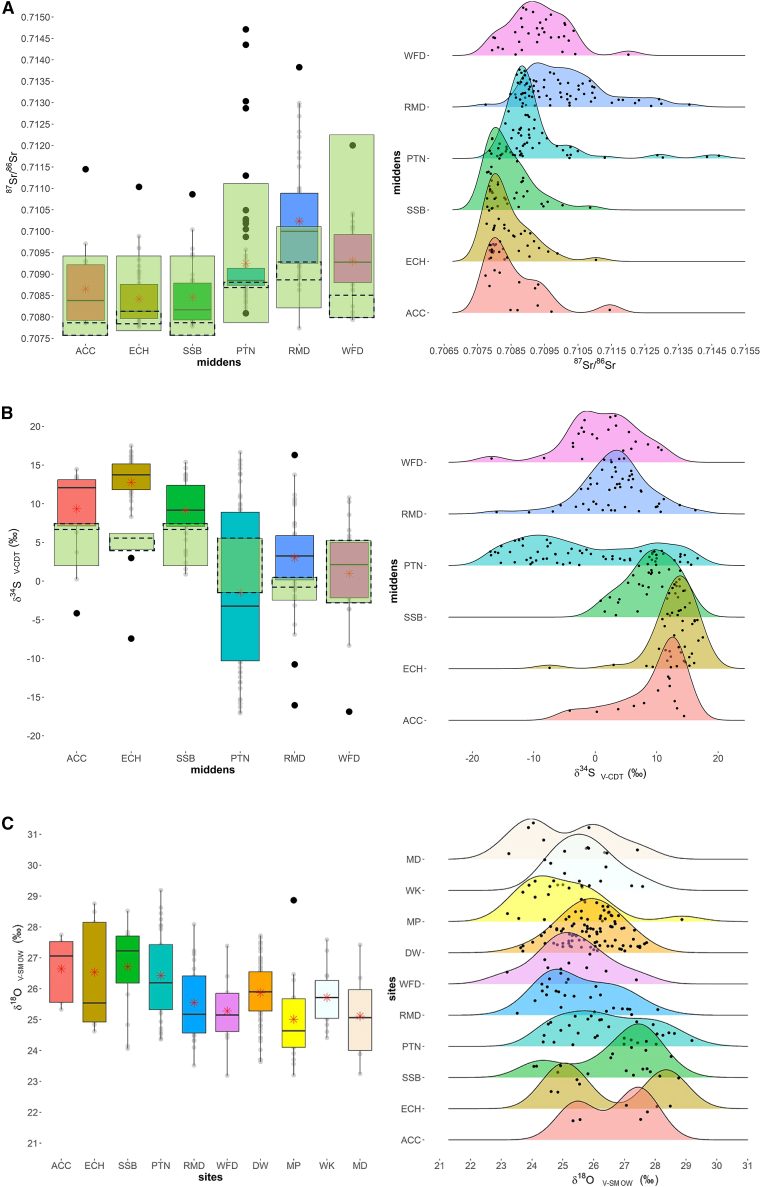
Box and whisker plots (representing 1.5 x IQR) and kernel density estimates (KDE) for the isotope proxies for all middens (A) Strontium (^87^Sr/^86^Sr), (B) Sulfur (δ^34^S V-CDT), (C) Oxygen (δ^18^O V-SMOW) values, also compared to Late Neolithic pigs from southern Britain. ACC = All Cannings Cross; ECH = East Chisenbury; PTN = Potterne; RMD = Runnymede; SSB = Stanton St Bernard; WFD = Wallingford. Late Neolithic sites: DW = Durrington Walls; MP = Mount Pleasant; WK = WKPE; MD = Marden. KDE visualize data distributions over a continuous interval, effectively providing a “smoothed” histogram. In boxplots A and B, the dashed rectangles are baseline ranges from primary plants analyses and green rectangles are baseline ranges based on BGS data^48^ and primary plants (British Geological Survey © UKRI 2023). Red stars indicate the mean. Late Neolithic data are taken from Madgwick et al.^39^

^87^Sr/^86^Sr and δ^18^O values combined with previously published δ^34^S, δ^15^N, and δ^13^C values^16^ are reported in Table S2. Summary statistics for ^87^Sr/^86^Sr and δ^18^O for each taxon are provided in Table S3. Table 2 reports ^87^Sr/^86^Sr, δ^18^O, δ^13^C, δ^15^N and δ^34^S statistics for all sites and ^87^Sr/^86^Sr, δ^34^S and δ^18^O values are presented graphically in Figures 2A–2C, the latter combined with a Neolithic pig sample for comparison. δ^34^S results are afforded brief summaries in this paper with fuller descriptions provided in Madgwick et al*.*^16^ In results descriptions, outliers are identified graphically (using the Quantile- Quantile [Q-Q] plots in the supplemental information) and highlighted in the text when they adhere to Tukey’s criteria (1.5x IQR).

**Table 2:** Summary statistics of ^87^Sr/^86^Sr (all samples) and δ^18^O (V-SMOW) (only pigs) values for the midden sites accompanied by δ^34^S (V-CDT), δ^15^N (AIR) and δ^13^C (V-PDB) values^16^

		ACC	ECH	SSB	PTN	RMD	WFD
^**87**^**Sr/**^**86**^**Sr**	n	18	39	38	71	53	33
	median	0.7083	0.7081	0.7082	0.7089	0.7100	0.7093
	mean	0.7086	0.7084	0.7085	0.7093	0.7102	0.7093
	min	0.7077	0.7078	0.7078	0.7081	0.7077	0.7079
	max	0.7114	0.7110	0.7109	0.7147	0.7138	0.7120
	IQR	0.0012	0.0008	0.0009	0.0004	0.0016	0.0011
	1 SD	0.0009	0.0007	0.0007	0.0013	0.0013	0.0009
**δ**^**18**^**O**_**c**_**(V-SMOW)**	n	5	9	15	29	25	10
	median	27.1	25.5	27.2	26.2	25.2	25.2
	mean	26.6	26.5	26.7	26.4	25.5	25.3
	min	25.3	24.6	24.1	24.4	23.5	23.2
	max	27.7	28.8	28.5	29.2	28.1	27.4
	IQR	2.0	3.2	1.5	2.1	1.8	1.2
	1 SD	1.1	1.8	1.4	1.4	1.2	1.2
**δ**^**34**^**S(V-CDT)**	n	15	38	36	60	49	23
	median	12.1	13.7	9.2	−3.2	3.3	2.1
	mean	9.4	12.8	9.1	−1.5	3.0	1.0
	min	−4.2	−7.4	0.9	−17.1	−16.1	−16.9
	max	14.5	17.5	15.4	16.7	16.3	10.8
	IQR	5.98	3.33	5.32	19.22	5.74	7.19
	1 SD	5.5	4.3	3.9	10.3	5.8	6.2
**δ**^**13**^**C(V-PDB)**	n	15	38	36	60	49	23
	median	−21.7	−21.9	−21.9	−21.7	−21.9	−22.0
	mean	−21.8	−21.8	−21.9	−21.7	−21.9	−22.1
	min	−22.6	−22.7	−22.5	−22.9	−23.2	−23.4
	max	−21.1	−20.9	−20.9	−20.5	−20.2	−21.4
	IQR	0.69	0.36	0.50	0.67	1.14	0.70
	1 SD	0.5	0.4	0.4	0.5	0.7	0.5
**δ**^**15**^**N(AIR)**	n	15	38	36	60	49	23
	median	5.5	4.9	5.8	6.2	6.2	5.8
	mean	5.5	5.0	5.9	6.1	6.4	5.9
	min	3.4	3.3	3.4	3.0	3.5	4.2
	max	8.3	7.8	10.1	9.0	8.9	7.8
	IQR	2.88	1.28	2.14	1.51	2.35	1.21
	1 SD	1.8	0.9	1.7	1.3	1.4	1.0

Midden sites: ACC = All Cannings Cross; ECH = East Chisenbury; PTN = Potterne; RMD = Runnymede; SSB = Stanton St Bernard; WFD = Wallingford.

#### ^87^Sr/^86^Sr summary

^87^Sr/^86^Sr ratio ranges are relatively wide but variable across midden sites. In Wiltshire, the three chalk sites, All Cannings Cross (*n* = 18), East Chisenbury (*n* = 39) and Stanton St Bernard (*n* = 38) have similar ranges (0.7077–0.7114, 0.7078–0.7110, 0.7078–0.7109 respectively), whilst Potterne (*n* = 71) shows a far wider range, incorporating more radiogenic values (0.7081–0.7147) – the broadest range of the entire dataset. All Cannings Cross, East Chisenbury, and Stanton St Bernard have similar means (ACC = 0.7086; ECH = 0.7084; SSB = 0.7085), whilst Potterne is higher (PTN = 0.7093), a pattern mirrored in standard deviations (ACC = 0.0009; ECH = 0.0007; SSB = 0.0007; PTN = 0.0013).

The kernel density estimates (KDE) (Figures 2A and S2) represent common patterns for the three Wiltshire sites with a high peak, and a skewed tail toward the only highly radiogenic value. By contrast, Potterne shows a multimodal distribution with a dominant peak and four subordinate peaks. At All Cannings Cross, East Chisenbury, and Stanton St Bernard, the most radiogenic values represent the only outliers (ACC01 = 0.7114; ECH12 = 0.7110; SSB05 = 0.7109). The Potterne dataset again shows a contrasting pattern, characterized by 13 outliers. Sites in the Thames Valley have relatively wide ranges. Runnymede (*n* = 53) represents the second largest range (0.7077–0.7138) only slightly narrower than Potterne. Wallingford (*n* = 33) ranges from 0.7079 to 0.7120. Runnymede shows the highest mean (0.7102), broadest IQR (0.0016) and SD (0.0013), with a completely different pattern compared to the sample distribution at Potterne (IQR = 0.0004, SD = 0.0013), the other site with a broad ^87^Sr/^86^Sr range. The KDE for Runnymede shows fewer distinct peaks with a very wide spread of values (Figures 2A and S2). Wallingford shows a similar pattern with a broad core of values, skewed by only one value. Both Runnymede and Wallingford have only one outlier (WFD11 = 0.7120; RMD51 = 0.7138).

Taxon-specific patterns are summarized in Table S3 and reported in the supplementary text.

#### δ^34^S summary

The δ^34^S data are described in detail in Madgwick et al.^16^ in the context of animal husbandry and only a shortened summary is provided here. δ^34^S values are exceptionally wide-ranging across the combined dataset, from −17.1‰ to 17.5‰. As for ^87^Sr/^86^Sr (and δ^13^C and δ^15^N), Potterne values were most diverse and accounted for almost the entire combined range (−17.1‰ to 16.7‰), with Runnymede’s range only slightly narrower (−16.1‰ to 16.3‰). These are among the widest site ranges globally in archaeological samples. Wallingford also has a substantial range, with a similar minimum value to nearby Runnymede (−16.9‰) but a markedly lower maximum (10.8‰). The Wiltshire chalk sites of All Cannings Cross, East Chisenbury, and Stanton St Bernard again have more limited, but still substantial, ranges with similar maximum values (14.5‰, 17.5‰ and 15.4‰ respectively) and also have moderately negative or very low positive minimum values (−4.2‰, −7.4‰ and 0.9‰ respectively). They contrast with other sites in being dominated by high (>10‰) values. There was limited inter-taxon variation at each site. However, it is noteworthy that the limited sample of caprines (*n* = 9) from Wallingford had a much wider range than other taxa at the site and the large sample of caprines from East Chisenbury (*n* = 20) had the smallest range with a dominance of high values (8.3‰–17.5‰).

#### δ^18^O summary (pigs only)

The δ^18^O isotope data provide a complex source of information due to issues surrounding climatic and dietary change and the nature of water sources exploited by pigs (see STAR Methods). The δ^18^O values from the Wiltshire middens show similarities to δ^34^S and ^87^Sr/^86^Sr. The three chalk sites have very similar means (All Cannings Cross: 26.6‰, Stanton St. Bernard: 26.7‰, East Chisenbury: 26.5‰) and variation in ranges is likely dictated by sample size. One noteworthy difference is the very large inter-quartile range at East Chisenbury (3.2‰), larger than all sites (including the Neolithic comparators, Figure 2C). The KDEs show similar patterns for these three Wiltshire sites with a multimodal distribution with two main peaks (Figure 2C). Another similarity is the diversity of values at Potterne (24.4‰–29.2‰), which has a wider range than all other sites and encapsulates almost all values from the other Wiltshire middens, as with δ^34^S and ^87^Sr/^86^Sr. In contrast to the other three Wiltshire sites, the KDE for Potterne shows a wide, continuous spread of values, comparable to Runnymede, which has the next widest range (23.5‰–28.1‰), as in other proxies. These patterns may be at least partially explained by the larger samples at these sites (Potterne *n* = 29; Runnymede *n* = 25). Wallingford also has a relatively substantial range (23.2‰–27.4‰); the KDE shows a high peak with no outliers. As would be expected given the W-E gradient in oxygen isotope values in water in the UK,^53^ values are lower at the Thames Valley sites (see Table 2; Table S3, means: Runnymede: 25.5‰, Wallingford: 25.3‰).

## Discussion

^87^Sr/^86^Sr ratios have long been a cornerstone of scientific research into mobility in archaeology. However, to gain a more comprehensive understanding, this analysis is increasingly combined with other isotope proxies, complementing and enhancing the interpretation of the data, as single isotope analysis only partially captures the complexity of past mobility.

The ^87^Sr/^86^Sr and δ^34^S data are often compared to isoscapes (maps of bioavailability) to distinguish local and non-local individuals. However, limitations in current methods continue to emerge (see limitations of the study and supplementary text). δ^34^S mapping, in particular, is still in its infancy.^54^ Although δ^34^S patterns across landscapes are becoming clearer in Britain,^48^ issues such as modern pollution, though significantly reduced,^44^ persist and therefore modern baselines must be used cautiously. Additionally, the variation in δ^18^O in water sources across landscapes cannot be easily mapped in relation to expected values in pig enamel (see below).

These issues are observable here, with substantial numbers of animals having values outwith the local range defined by primary plant analysis and the published British biosphere map.^48^ Generally, outliers highlighted in the results broadly align with those outwith the biosphere range. However, all sites except Potterne (with 13 outliers) and Wallingford show a greater number of non-locals than the statistically defined outliers (despite conservative regional ranges being defined). If the primary plant samples are considered separately, then all midden sites have a majority of non-local animals. This likely indicates that the primary sampling was not extensive enough and therefore, some locally raised animals are outside the defined local range. This is to be expected given the limits of the plant sampling strategy – it is highly likely that some animals acquired food beyond the immediate vicinity of the site even if they were locally raised (e.g., seasonal pasturage exploitation, woodland resources for pigs).

The use of multiple isotope proxies mitigates limitations inherent in single-isotope approaches.^39^^,^^47^^,^^55^^,^^56^^,^^57^^,^^58^ This approach addresses some issues of equifinality and ensures that samples appearing local on one proxy can still be identified as non-local using other isotope systems. Therefore, we discuss faunal samples from different middens using a multi-proxy approach, offering a more nuanced understanding of mobility and husbandry practices across midden sites. Primary plant values and biosphere maps are used as a supplementary source of evidence for origins, particularly for outlying individuals. In addition, issues with using δ^18^O drinking water maps for assessing origins are combatted by through comparisons with existing pig δ^18^O data,^39^ analyzed using the same methodology.

### Mobility in Wiltshire midden sites

**Figure 3:**
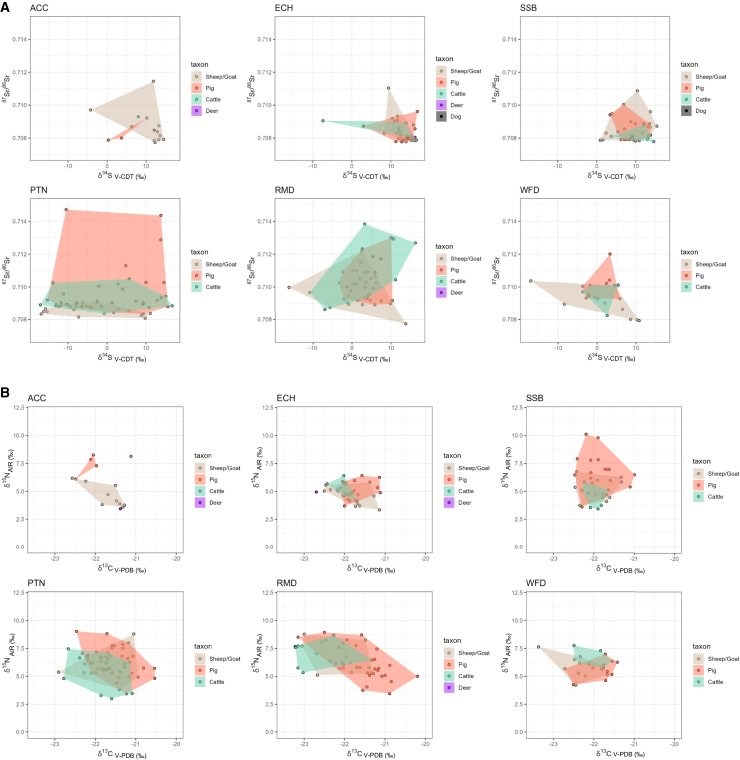
Convex-hull-bivariate scatter plots comparing the isotope proxies across taxa at all middens (A) Strontium (^87^Sr/^86^Sr) and sulfur (δ^34^S V-CDT), (B) Carbon (δ^13^C V-PDB) and nitrogen (δ^15^N AIR). ACC = All Cannings Cross; ECH = East Chisenbury; PTN = Potterne; RMD = Runnymede; SSB = Stanton St Bernard; WFD = Wallingford. A convex hull is a polygonal shape that encapsulates all the individuals for each taxon.

**Figure 4:**
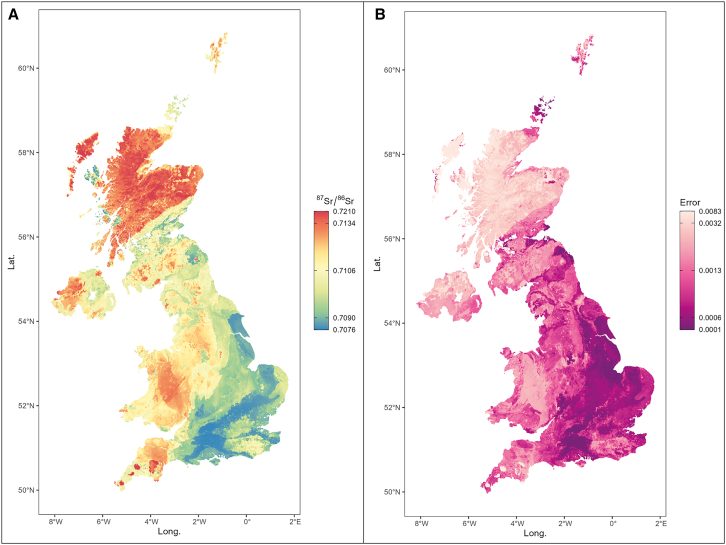
Random forest ^87^Sr/^86^Sr isoscape and spatial uncertainty maps based on FEASTNET plant data and BGS bioavailable data Maps were produced following the protocol of Bataille et al.^59^ (see STAR Methods) and data from.^48^ Contains OS data © Crown Copyright and database right 2020 © and British Geological Survey materials © UKRI 2023.

**Figure 5:**
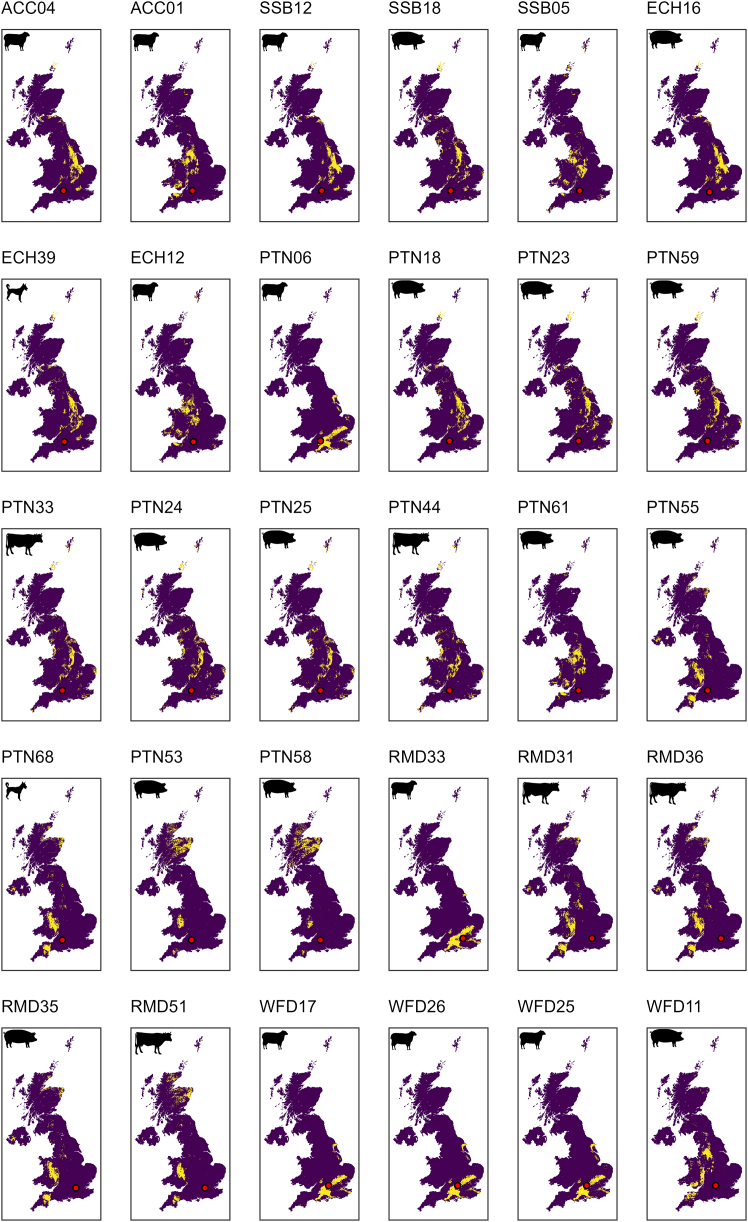
^87^Sr/^86^Sr geographic assignment of non-local individuals Map indicating likely non-local individuals’ provenance. Maps are filtered according to the top 5% likely provenance (yellow pixels). Non-local individuals are selected based on Tukey’s criteria (1.5x IQR) and outliers outside the estimated local range (see STAR Methods).

Wiltshire sites show variable patterns of faunal mobility. All Cannings Cross, East Chisenbury, and Stanton St Bernard show a narrow range in both ^87^Sr/^86^Sr and δ^34^S (Figure 3A), hinting at animals raised locally with some exceptions. Plant ^87^Sr/^86^Sr data support local origins, but plant δ^34^S suggests the majority are non-local, though this is almost certain to be an issue with modern plants not directly correlating with archaeological δ^34^S (see supplementary text). This is supported by the presence of a cervid with a much higher value (16.2‰) than the plants at East Chisenbury, suggesting a higher baseline for chalk sites in the past. The pig δ^18^O dataset is more wide-ranging at these sites and, unlike other provenancing proxies, does not differ markedly from Potterne. This is highly likely to relate to more diverse management regimes of pigs at middens, with dietary diversity impacting the δ^18^O range, rather than diverse origins (see supplementary text). Potterne does, however, exhibit the widest δ^18^O range (as in ^87^Sr/^86^Sr and δ^34^S), again pointing to a wider catchment. The interpretative potential of this smaller pig-only dataset is more limited and used as a supplementary source of evidence only. Overall, Potterne data evidence a substantial number of animals across species being raised beyond the vicinity of the midden and brought to the site for feasting events. The breadth of results also indicates a substantial catchment comprising multiple regions – this is not a pattern of one or two major production centers supplying the feasts with animals (see Figures 4 and 5). This is supported by the δ^13^C and δ^15^N results,^16^ which demonstrate highly variable pasturage and management strategies (Figure 3B) and by the δ^18^O data which suggest pigs from both eastern and western zones of Britain and/or variable management regimes are represented.

Focusing on outliers as a proxy for non-local origins is problematic, as large numbers of animals may come from wide-ranging locations. This is likely the case at Potterne, as although the local biosphere range is relatively wide, it cannot account for the breadth of δ^34^S values (that show no outliers), which is among the widest published globally. Some clear inter-taxon patterns in mobility are observable at Potterne. Caprines have a narrow ^87^Sr/^86^Sr range with one outlier (PTN06 = 0.7081; see Figures 4 and 5) and dominance of lower (<0.709) ratios suggestive of husbandry on chalklands. However, the δ^34^S range is broad and does not overlap with other Wiltshire middens (which have a more local catchment), suggestive of a markedly broader and very different catchment for caprines at Potterne. There is no evidence that this catchment includes exposed coastal areas given the absence of values > 11‰ (Figure 3A, see^60^) and, surprisingly, the local Wiltshire chalkland is also likely not represented. The prevalence of negative δ^34^S values demonstrates many animals across species raised in wetland and/or Jurassic clay environments.^61^^,^^62^ Given the complexities of δ^34^S biosphere mapping and the lowland zone that the site overlooks, it is plausible that some animals may have been raised in the locality, though there is no clear evidence that this was a wetland in prehistory.^25^ However, the range of negative values suggests more than one wetland location (but a single wetland region with a wide-ranging baseline cannot be entirely discounted, see^44^). The only Potterne non-local caprine ^87^Sr/^86^Sr outlier might have been raised near the site, in contrast to the other Wiltshire sites’ non-local caprines (see below and Figures 4 and 5). The provenance map indicates a higher probability in the south of England (see Figures 4 and 5).

The wide ^87^Sr/^86^Sr range, with numerous outliers in pigs and some in cattle, is particularly noteworthy at Potterne (Figure 3A). While the very broad δ^34^S range for the three domesticates could suggest that the immediate vicinity of Potterne has wide bioavailable δ^34^S, the diverse ^87^Sr/^86^Sr (supported by δ^18^O) ranges unquestionably point to pigs brought from wide-ranging areas, likely coastal, inland and wetland zones, from broad lithologies and from eastern and western areas (and/or having been subject to diverse management strategies). Pig outliers show at least four main provenance groups, defined by ^87^Sr/^86^Sr probabilistic geographic assignment (see Figures 4 and 5, Groups #1: PTN18, PTN23, PTN59, PTN24, PTN25; #2: PTN61, #3: PTN55; #4: PTN53, PTN58). Each of these groups has variable δ^34^S values, indicating diverse wetland, coastal and inland environments, again confirming different origins of pigs brought to Potterne for feasting events. Two outlying highly radiogenic values (>0.714; Group 4) are from two different areas, one coastal and one wetland based on δ^34^S. The southwest peninsula has coastal areas, highly radiogenic granitic lithology and wetland zones and, therefore, would seem a likely origin, but the δ^18^O isotope values suggest otherwise, with these pigs having the lowest values in the dataset, thus pointing to origins in eastern Britain. Given the paucity of radiogenic lithology in eastern Britain, Scotland cannot be discounted (see Figures 4 and 5). Lead (Pb) isotope data would be required to confirm this^48^^,^^63^ given that δ^18^O should not be fore-fronted in interpretation due to the potential dietary impact on variation. However, there are issues concerning whether Pb is coeval with other isotope systems, so this must be considered with caution.^64^ A similar picture is also true for outlying cattle with similar ^87^Sr/^86^Sr ratios but very different δ^34^S, reconfirming the wide catchment of fauna brought to Potterne for feasting.

Overall, whilst non-local animals were present across taxa, they were clearly most common among pigs and cattle (see Figures 4 and 5). Pigs were brought from more diverse areas and, in some instances, over substantial distances, to Potterne. Pigs are generally more common at midden sites than in later prehistoric settlements, and Wallingford, Runnymede and Potterne all have high percentages of pigs (>28% NISP of the main food domesticates^13^). Pigs are frequently considered a sign of wealth and were likely brought to the site for feasting events, imbued with a degree of significance, given that cattle and caprines dominate faunal assemblages across later prehistoric Britain.^13^^,^^39^ There are many advantages to using pigs as feasting animals. Pigs have a short gestational period, can have multiple litters in a year, providing numerous piglets and reach prime meat weight quicker than caprines and cattle. As omnivores, pigs can be raised on wide-ranging resources, including food scraps, and can efficiently convert local resources to meat in different environments. Pig meat cures particularly well due to intrinsic characteristics of the flesh (e.g., fat content), making it suited for preservation, storage and transport.^65^ Even though transporting pigs over distances requires considerable effort, examples are attested in ethno-archaeological studies,^66^ but this certainly represents a considerable investment of effort at Potterne.

East Chisenbury, a midden of monumental proportions, appears to be dominated by locally raised animals, with a narrow range in both ^87^Sr/^86^Sr and δ^34^S (Figure 3A), and a relatively limited range in dietary isotope proxies.^16^ Outliers comprise a caprine (ECH12 = 0.7110) with high ^87^Sr/^86^Sr, though the ratio is markedly lower than outlying pigs and cattle at Potterne, and cattle and pig individuals with lower δ^34^S values (−7.4‰ and 3.0‰), again unremarkable in the context of Potterne. The negative cattle δ^34^S value suggests wetland origins but the other values are undiagnostic. Caprines were overwhelmingly dominant at East Chisenbury, accounting for well over half of all specimens^19^ and that there is strong evidence for sheep dairying,^18^ wool and meat production.^19^ However, the intensive, multi-modal caprine economy appears to have been dominated by local animals, rather than the site being a hub for animals from elsewhere to provide these products.

Data suggest a relatively rigid management strategy utilizing local landscape resources, potentially as part of a symbiotic strategy with All Cannings Cross and Stanton St Bernard, more modest middens with a similar pattern of isotope values and only a single outlier, a caprine with high ^87^Sr/^86^Sr (ACC01 = 0.7114) at All Cannings Cross and one at Stanton St Bernard (SSB05 = 0.7109), with very similar values and provenance estimation to East Chisenbury caprine outlier (ECH12; see Figures 4 and 5). This again points to similarities in the three Wiltshire sites, also in terms of outliers, with similar ^87^Sr/^86^Sr as well as δ^34^S values. Nonparametric pairwise comparisons between middens support these patterns: ^87^Sr/^86^Sr ratios show that the three midden sites of East Chisenbury, All Cannings Cross and Stanton St Bernard do not have a statistically different sample distribution (Table S4), whereas Potterne, despite its proximity to the other Wiltshire sites and relatively similar ^87^Sr/^86^Sr local baseline range, is significantly different from the other three Wiltshire middens. By contrast, δ^34^S distributions are significantly different among the Wiltshire middens (with the exception of All Cannings Cross vs. Stanton St Bernard). The most noteworthy difference between the three sites is the more wide-ranging δ^15^N in pigs from Stanton St Bernard, demonstrating more diverse management practices, with more evidence for omnivorous diets^16^(Figure 3B).

### Mobility in the Thames Valley midden sites

Thames Valley sites contrast with the Wiltshire middens, though one site again shows a far wider catchment. Overall, animal management regimes seem less controlled than at All Cannings Cross, Stanton St Bernard, and particularly East Chisenbury, as also shown by the pairwise nonparametric tests (see Table S4). Runnymede shows very wide ^87^Sr/^86^Sr, δ^18^O and δ^34^S ranges, wider than all sites except Potterne in these proxies, providing strong evidence for a broad catchment. In contrast to Potterne, domestic herbivores have broader ranges in δ^34^S and ^87^Sr/^86^Sr than pigs at Runnymede, with cattle showing the widest ^87^Sr/^86^Sr range despite having fewest samples (*n* = 11). Cattle may have been the key feasting animal at Runnymede, imbued with special status and therefore often brought from a much wider catchment (as with pigs at Potterne). However, there is no evidence that they were subject to prescribed management regimes for the feast, as δ^13^C and δ^15^N ranges were broadly comparable to caprines^16^ (Figure 3B).

A caprine and a cattle individual have outlying negative δ^34^S values (−16.1‰, cattle; −10.8‰, caprine) indicative of wetland origins. Values slightly below 0‰ may be found locally (as indicated by a cervid), but more extreme negative values such as these have been produced in animals from western parts of the Thames in Oxfordshire,^67^ an area consistent with the relatively undiagnostic ^87^Sr/^86^Sr (0.7097–0.7010). In addition, an outlying cattle sample (RMD31) with a coastal δ^34^S value (16.3‰) has a ^87^Sr/^86^Sr ratio (0.7127) ruling out coasts in southern and eastern England and therefore representing long distance movement from western coasts (see Figures 4 and 5). Although pigs are not as wide-ranging as herbivore domesticates, their values are still diverse across proxies and are not consistent with all being locally raised. This is emphasized by the δ^18^O data, which has a comparable absolute range to Potterne, but lower values, perhaps pointing to a predominantly regional catchment in the east of England. The diverse δ^13^C and δ^15^N values for pigs support this and highlight that diverse management strategies and foddering regimes (likely including forest resources and meal waste) were employed to raise meat for feasting events at Runnymede. Overall, Runnymede shows a similar pattern to Potterne in being a hub for regional networks, with some animals, particularly herbivores, coming from far further afield. Some cattle, with more radiogenic values, are likely to have more western and northern origins, demonstrating widespread catchments (see Figures 4 and 5).

Wallingford did not command the same catchment as Runnymede, but nor did the site show an intensive, specialized, locally focused faunal economy, as was evident at the Wiltshire chalkland sites. This was also evidenced by pairwise nonparametric tests between Wallingford and Runnymede for ^87^Sr/^86^Sr (Table S4). Cattle and pigs have a relatively limited range in both ^87^Sr/^86^Sr and δ^34^S and sit close to the core of the Runnymede range, suggesting relatively locally raised animals (Figure 3A). This is clear in the δ^34^S data, where pig (−3.6‰ to 5.9‰) and cattle (−3.6‰ to 5.5‰) ranges align closely with the baseline range (−3.2‰ to 5.2‰). Evidence for non-local animals based on Tukey’s method of identifying outliers and estimated local ranges (see STAR Methods) includes a pig with a more radiogenic ^87^Sr/^86^Sr ratio (0.7120), likely from western areas of Britain based on a high δ^18^O value (26.4‰) (see also Figures 4 and 5) and three caprine individuals with low (≤0.7080) ^87^Sr/^86^Sr ratios, suggestive of chalkland origins, likely in southern England (see also Figure 5). The pig has a high δ^18^O value, suggesting origins in western Britain.

Caprines have a wider range in δ^34^S than other taxa, comparable to Runnymede, suggesting they were drawn from a wider catchment, including from wetland zones as at Runnymede, perhaps from the western Thames Valley (Figure 3A). Only a single animal has a high (>14‰) δ^34^S value from the Thames Valley (a cattle individual from Runnymede). This indicates animals were very rarely sourced from the exposed western coasts of Britain and provides further support for a wider regional network focused on eastern England, with some animals having origins far further away. The higher δ^15^N values in herbivores in the Thames Valley compared to Wiltshire^16^ also points to predominantly regional networks (with lower landscape baselines in the Wiltshire chalklands). Runnymede clearly had a wider catchment and had the role of a regional hub in the Thames Valley.

### The role of middens at the Bronze Age-Iron Age transition: Regional comparisons

A PCA incorporating δ^13^C, δ^15^N, δ^34^S, ^87^Sr/^86^Sr datasets demonstrates the difference between the group characterized by wider variability (Potterne, Runnymede and to a lesser extent Wallingford) and the group of the remaining Wiltshire middens which show similar, limited variability (see Figure S4). It is noteworthy that Wallingford and Potterne, despite the distant locations, show a non-significant difference in both ^87^Sr/^86^Sr and δ^34^S (see Table S4) while the shape of the distribution (see Figures 2A and 2B) indicate a different pattern of variability. This once again shows that All Cannings Cross, East Chisenbury, and Stanton St Bernard were raising animals locally, following a prescribed management regime, while Potterne, Runnymede and, to a lesser extent, Wallingford were central hubs where animals were brought in for feasting events from distant locations. Given the lack of significant differences, it is possible that Wallingford and Potterne exploited similar production zones or even exchanged animals, linking these regional networks.

### Limitations of the study

This study presents a number of limitations that need to be taken into consideration. First of all, there are limitations regarding the number of samples considered for each midden site. Nonetheless, by considering all six midden sites, this represents the largest multi-isotope dataset on faunal remains yet generated in archaeology. As highlighted in the main text, there are limitations in ^87^Sr/^86^Sr and δ^34^S mapping (see also supplementary text). Primary plants collected for the study were sampled just from within the vicinity of each site, within approximately 5 km. This can lead to a misrepresentation of the real number of outliers, pointing to more non-local individuals than are really present. However, it must be taken into account that producing an exhaustive map of bioavailability is expensive and time-consuming. Published biosphere maps can also suffer from a limited sample density. ^87^Sr/^86^Sr biosphere maps for the UK are more advanced with substantially more samples than other biosphere maps such as δ^34^S. The δ^34^S isotope biosphere map presented by Evans et al.^48^ is much more accurate than previous iterations, showing more clear patterns on wetlands —which tend to produce negative values^44^— and estuaries which can generate marine-affected values inland.^48^ However, issues of modern pollution persist, making it impossible to compare modern plant with archaeological δ^34^S values. Furthermore, δ^34^S cycling and its impact on bioavailability over time is poorly understood, as the effect of underlying geology. Further limitations to the study relate to δ^18^O mapping (see supplementary text). δ^18^O variation in water sources across the landscape cannot easily be mapped and compared to the expected values in pig enamel. For these reasons, we have compared midden data in the context of a substantial dataset of Late Neolithic pigs from southern Britain.

### Summary and conclusion

Middens have been interpreted as the outcome of the coming together of vast numbers of people and animals for feasts. This study, the largest multi-isotope faunal dataset yet delivered in archaeology, has demonstrated that, despite their structural similarities, middens had diverse roles. Some middens anchored wide-ranging regional networks, whilst others drew from a more local to regional catchment. Given the proximity of all middens to rivers, it is likely that waterways played a role in the movement of people, objects and livestock, as evidenced elsewhere in LBA-EIA Britain.^68^ Most middens showed an agile approach to animal management, with diverse approaches to maximizing productivity in terms of the range of pasturage, manuring and foddering that was employed. Others evidenced a more specialist, standardized economic role, with an intensive, single species mixed economic strategy to maximize the productivity of the local environs of the site.

In Wiltshire, East Chisenbury and Potterne’s multi-isotope data demonstrate very different roles. The wide-ranging data across all isotopes at Potterne evidence large numbers of animals raised in diverse locations across southern Britain and beyond, subject to different management strategies, pointing to a broad catchment to intensify production and raise large amounts of meat for feasting. Pigs constitute the highest number of non-local individuals and were brought from diverse areas, demonstrating the powerful draw of feasts. The site represented an important lynchpin in the landscape and these feasting events supported a substantial network across the region and beyond. By contrast, East Chisenbury is dominated by locally raised animals with a relatively rigid management strategy. Zooarchaeological and multi-isotope data suggest a site based on sheep dairying, wool and meat production dominated by locally managed caprines. A similar pattern of management is represented in the more modest assemblages of All Cannings Cross and Stanton St Bernard, represented by locally raised animals and strict management. These sites appear to have pursued an intensive, mixed economic strategy specialized on caprines and maximizing the productivity of the local chalkland zone. These greatly contrasting social and economic responses to the upheaval at the end of the Bronze Age are surprising given the proximity of the sites.

In the Thames Valley, Runnymede, as Potterne, shows strong evidence for a broad catchment with domestic herbivores showing more varied values than pigs. This site, and the feasts that took place here, supported a broad network, principally focusing on south eastern England but with considerable numbers of animals (and thus people) coming from further afield. As with pigs at Potterne, cattle may have been the key feasting animal at Runnymede and was often brought over much greater distances. Wallingford did not show a wide catchment as Runnymede, but nor did the site show an intensive, specialized, locally focused faunal economy, as was as evident at East Chisenbury, All Cannings Cross and Stanton St Bernard. It seems rather that Wallingford operated in a similar way to Runnymede, but at a smaller scale, perhaps even as a linked, subsidiary site, supporting a regional network with animals subject to a more limited range of management regimes.

This study demonstrates the great value of a multi-isotope approach combined with biosphere sampling in providing greater resolution on site catchments, networks of supply and animal (and human) mobility. It highlights the requirement of multiple isotope proxies, even to establish the likely local vs. non-local status of animals with confidence and the potential to move beyond this dichotomy and refine origins by combining proxies relating to both diet/management and origins. However, it also demonstrates that ambiguities still persist, even with extensive, multi-factorial datasets and identifying origins with precision frequently remains beyond the potential of multi-isotope analysis. An improved understanding of sulfur isotope variation in the British biosphere may lead to further interpretative refinement in the future. Overall, the research points to the dynamic networks that were anchored on feasting events during this period and the different, perhaps complementary, roles that different middens had at the Bronze Age-Iron Age transition.

## Resource availability

### Lead contact

Further information and requests for resources and reagents should be directed to and will be fulfilled by the Lead contact, Richard Madgwick (MadgwickRD3@cardiff.ac.uk).

### Materials availability

This study did not generate new unique reagents.

All data produced in this study can be found in Tables S1 and S2 in this paper and Supplementary Table 1 in Madgwick, Esposito and Lamb.^16^

The fauna remains from the six midden sites are stored at the Wiltshire Museum, the Oxfordshire Museums Service and the British Museum. These museums, which hold the legal responsibility for the collections from the midden sites of All Cannings Cross, East Chisenbury, Potterne, Stanton St Bernard (The Wiltshire Museum), Runnymede (The British Museum) and Whitecross Farm, Wallingford (Oxfordshire Muesums Service), have authorized their study and isotope analysis.

### Data and code availability

General archaeological information (i.e., context number), taxon (i.e., Sheep/Goat, Pig, Cattle, Dog, Cervidae), and type of sample analyzed (e.g., bone and/or teeth) are reported in Table S2 of this article.

• Data: ^87^Sr/^86^Sr and δ^34^S isotope values from environmental samples are available in Table S1 of this article.
• ^87^Sr/^86^Sr data from all faunal remains and δ^18^O data of pig samples of the midden sites are available in Table S2 of this article.
• δ^34^S, δ^15^N, δ^13^C data of fauna samples are available in Table S2 of this article and Supplementary Table 1 of Madgwick, Esposito and Lamb.^16^ Any additional information is available from the lead contact upon request.
• Code: No code generated in this study.

See key resources table for more information.

## Acknowledgments

We are grateful to 10.13039/501100000267AHRC, UKRI, and the 10.13039/501100000780European Commission for funding (see funding statement). Nathaniel Harrop-Pender, Jerome Hancock, and Hugh Nianias assisted with some sample preparation and Kirsty Harding produced Figure 1. We are also grateful to Dickie Bennett, Richard Osgood, and the military veterans who helped with sample processing on the project as part of the Breaking Ground Heritage/Operation Nightingale program. We would like to thank Luca Bondioli for facilitating graph production and statistical analysis and Hongjiao Ma for processing plants from East Chisenbury. We are very grateful to Lisa Brown (The Wiltshire Museum), Angie Bolton (Oxfordshire Museums Service), Neil Wilkin and Sophie Crump (British Museum) for providing access to collections and sampling support.

This research was funded by the 10.13039/501100000267Arts and Humanities Research Council (AH/T006528/1) and 10.13039/100014013UKRI (CoA scheme). Carmen Esposito was supported by the European Union's Horizon Europe Research and Innovation programme under the Marie Skłodowska-Curie Actions PF (GA no. 101065320 — TULAR) during manuscript writing and revision stage. The graphical abstract was created with BioRender.com.

## Author contributions

Conceptualization, R.M. and A.L.L.; methodology, C.E., R.M., A.L.L., and F.L.; investigation, C.E., R.M., and A.L.L.; visualization, C.E. and F.L.; supervision, R.M.; writing – original draft, C.E. and R.M.; writing – review and editing, all authors.

## Declaration of interests

The authors declare that they have no competing interests.

## STAR★Methods

### Key resources table

**Table:** 

REAGENT or RESOURCE	SOURCE	IDENTIFIER
**Deposited data**		

Raw and analyzed data	This study	Tables S1 and S2
Raw and analyzed data	Madgwick, Esposito and Lamb^16^	Table S1

**Software and algorithms**		

R language and environment for statistical computing (ver. 4.4.2)	R Core team 2024	https://www.r-project.org/

### Experimental model and study participant details

#### Archaeological faunal samples

A total of 254 samples were analyzed for the FEASTNET project, including previous analysis.^16^ A total of 252 individuals, principally caprine (*n* = 86), pig (*n* = 93) and cattle (*n* = 52), augmented by deer (*n* = 4) and dogs (*n* = 17) from six middens, were analyzed for ^87^Sr/^86^Sr analysis. All sheep/goat samples are defined as caprine. The majority could not be identified to exact species but those that could were all sheep. Two of the 254 samples (RMD47 and WFD08) had insufficient enamel for ^87^Sr/^86^Sr and δ^18^O analyses. Samples are reported in Table S2 with details of the taxa, elements, and contexts sampled; samples that did not meet quality control criteria for δ^13^C, δ^15^N and δ^34^S are highlighted in gray. Samples only analyzed for ^87^Sr/^86^Sr are highlighted in blue.

#### Modern plant samples

Efforts were made to sample plants for biosphere characterization from areas that had limited anthropogenic impact at the six midden sites. Plant samples were collected in paper bags (*n* = 16 see Table S1) and freeze-dried, coarsely crushed to tea leaf consistency, and the three plants were homogenized to provide a single sample and weighed to approximately 300 mg per sample. The plant samples were not washed or cleaned prior to analysis with the aim of reproducing the natural biosphere.

#### Ethical statements and additional information

This research study was carried out in compliance with the relevant regulations for the treatment of remains from archaeological contexts, as outlined in the International Council of Archaeozoology (ICAZ) for the treatment and the destructive sampling of archaeofaunal material.

### Method details

#### ^87^Sr/^86^Sr faunal tooth enamel and plants samples

The analysis of ^87^Sr/^86^Sr ratios provides data useful for exploring human and faunal mobility based principally on lithological settings where individuals acquire food during the development of the dental arcade (see^69^). Generally, variation is dictated by the age of the geological formations (with older lithologies providing higher ratios) and the Rb/Sr content of the rock. This is a gross oversimplification of the complex drivers of variation and more detailed summaries are provided elsewhere.^40^^,^^70^

Initial sample preparation for ^87^Sr/^86^Sr analysis was undertaken at Cardiff University BioArchaeology (CUBA) laboratory. Enamel samples (c. 20–40 mg) were cut from teeth using a diamond saw and burr to remove any adhering contaminants and at least 10 μm of enamel surface and all dentine. The sampled enamel surface was not standardised. Surfaces were targeted based on preservation and ease of sampling. Narrow (c. 2 mm) strips of enamel were extracted to limit the duration of Sr incorporation and thus reduce the chance that different biosphere signals were averaged in the sample. Due to sample availability, a range of different teeth at different wear stages were sampled and therefore locations on the cusp were also not standardized. Wherever possible, for early developing teeth (such as M1s), enamel was sampled close to the root-enamel junction (REJ) to ensure a post-weaning signal (as pigs were also sampled for δ^18^O). Earlier forming enamel (close to the occlusal surface) was sampled for later developing teeth (M2 and M3) to provide an earlier life (but post-weaning) signal for origins. Enamel samples were cleaned in an ultrasonic bath in deionised water and dried down.

Plant samples were transferred into a fume hood of a clean lab (laminar flow class 100) to be dissolved in 9 mL of distilled concentrated HNO_3_ and 1 mL of concentrated H_2_O_2_ in TFM pressure vessels. The vessels were capped and transferred into a Milestone Ethos Easy microwave digestion system, where they were digested under high temperature and high pressure using a preset standard environmental plant sample digestion protocol. Then the plant samples were transferred into PMP beakers in to dry down before undergoing the same chemical preparation protocol described as enamel samples.

Sample chemistry was conducted at the Cardiff Environmental Laboratory for Trace element and Isotope Chemistry (CELTIC). Enamel samples were digested overnight in 8 M HNO_3_ on a hotplate at 120°C. Plant leaves were placed in distilled HNO_3_ and H_2_O_2_ and digested using the Milestone Ethos Easy microwave digestion system. The sample was spun down using a microcentrifuge to isolate residue and the supernatant transferred into Savillex beakers. Following preparation, enamel and plant samples followed the ^87^Sr/^86^Sr extraction protocol in Scorrer et al..^71^ Approximately 100 μL of pre-cleaned Eichrom Sr-Spec resin is loaded into extraction columns. Digested samples were then loaded into the resin columns, Matrix elements, including Ca and trace Rb, were removed in several washes of 8 M HNO_3_ before Sr was eluted and collected in 0.05 M HNO_3_. Samples dried overnight on a hotplate at 120°C, then the process is repeated for a second time for the effective removal of any remaining traces of Ca. Once dry, purified samples were re-dissolved in 0.3 M HNO_3_.

The ^87^Sr/^86^Sr ratios were measured using a Nu Plasma II multi-collector inductively coupled plasma mass spectrometer (MC-ICP-MS) at Cardiff University. Samples were introduced using an Aridus II desolvator introduction system. All data were first corrected for on-peak blank intensities, then mass bias corrected using the exponential law and a normalization ratio of 8.375209 for ^88^Sr/^86^Sr.^72^ Residual krypton (Kr) and rubidium (^87^Rb) interferences were monitored and corrected for using ^82^Kr and ^83^Kr (^83^Kr/^84^Kr = 0.20175 and ^83^Kr/^86^Kr = 0.66474; without normalization) and ^85^Rb (^85^Rb/^87^Rb = 2.5926), respectively. Analysis of NIST SRM 987 during the analytical session gave a ^87^Sr/^86^Sr value of 0.710292 ± 0.000007 (2σ *n* = 11), and all data are corrected to NIST SRM 987 values of 0.710248.^73^ Total procedural blanks are typically less than 20 pg of Sr, which is negligible relative to the Sr in samples (greater than 20 ng). Accuracy of the NIST SRM 987 normalization and the chemistry processing was assessed by repeat measurements of ^87^Sr/^86^Sr ratio in NIST SRM 1400 (Bone Ash, processed through chemistry similar to the unknown samples), giving an average ^87^Sr/^86^Sr ratio of 0.713111 ± 0.000014 (2σ *n* = 5), which is consistent with the published value (0.713126 ± 0.000017).^74^

#### δ^18^O (pigs) tooth enamel

δ^18^O analysis represents a complex source of data for exploring origins and mobility. The approach is commonly used to examine lifetime seasonal mobility and management in cattle, caprines (e.g.,^75^^,^^76^) and occasionally pigs,^77^ but this requires many analyses per individual and is beyond the remit of this study (which focuses on animal origins, rather than in-life mobility). Whilst δ^18^O values in the long-crowned hypsodont molars of cattle and caprines are highly seasonally affected, this is not the case for pig molars, which have been shown to provide consistent values.^78^ Therefore, single analyses have the potential to provide useful information on origins and δ^18^O isotope analysis was therefore undertaken for pigs only. By sampling enamel that develops at approximately the same time any remnant seasonal effect will be minimised (as the chance for double farrowing having happened in this phase of prehistory is remote). In addition, later developing (REJ) enamel was targeted from early developing teeth wherever possible to minimise any remnant nursing signal. There is growing evidence that relatively minor differences in sampling, pre-treatment and mass spectrometry can impact on δ^18^O isotope values and this has the potential to be a major problem in single sample analysis such as this (e.g.,^79^^,^^80^). Therefore, values from midden pigs are compared to a substantial sample (*n* = 131) of Late Neolithic pigs from southern Britain (Figure 2C) that were analyzed in the same laboratory using the same methodology.^39^ These data are used with caution and as supplementary evidence for mobility as the use of δ^18^O data in this way remains exploratory and dietary regimes in omnivores such as pigs could impact on variation.

The δ^18^O analyses were performed on pig enamel samples (*n* = 93; see Table S2). following the protocol described in.^71^ Enamel was powdered using an agate pestle and mortar at CUBA. The isotope composition of the structural carbonate within the enamel was measured (δ^18^O_carbonate_). At the Cardiff University Stable Isotope Facility, samples were acidified for 5 min with greater than 100% *ortho*-phosphoric acid at 70°C^79^ and analyzed in duplicate using a Thermo MAT253 dual inlet mass spectrometer coupled to a Kiel IV carbonate preparation device. The resultant isotope values are reported as per mil (^18^O/^16^O) normalized to the V-PDB scale using an in-house carbonate reference material (BCT63) calibrated against NBS19 certified reference material. The δ^18^O carbonate values are then converted into the V-SMOW scale (δ^18^O_V-SMOW_ = 1.0309 x δ^18^O_V-PDB_ + 30.91^81^). The long-term reproducibility for δ^18^O BCT63 is ±0.03‰ (1σ). The standard deviation of replicate δ^18^O measurements is 0.049‰. The δ^18^O_carbonate_ values were converted to the expected δ^18^O phosphate (δ^18^O_p_) values (following^82^) to allow for comparison with other datasets. The error on the calculated δ^18^O_p_ values is 0.24‰, based on the analytical error and the error in the conversion regression equation.

#### δ^34^S plant samples

The same plants collected for ^87^Sr/^86^Sr were analyzed to characterize the modern δ^34^S biosphere of midden sites (*n* = 16; see Table S1), following.^44^ About 1 g was transferred to a cryogenic mill where they were reduced to a powder over 1–2 min. For δ^34^S analysis 2 mg of powdered material was weighed into tin capsules and measured in duplicate by continuous flow-elemental analyser-isotope ratio mass spectrometry (CF-EA-IRMS) at the British Geological Survey, Keyworth UK. The instrumentation comprises a Thermo Fisher EA IsoLink coupled to a Delta V Plus IRMS via a ConFlo IV interface. *δ* ^34^S isotope ratios (*δ*^34^S) are reported in per mil (‰) and normalized to Vienna Canyon Diablo Troilite (V-CDT) using the International Atomic Energy Agency (IAEA) reference materials IAEA-S-1 (silver sulfide, *δ*^34^S_V-CDT_ = −0.30‰). IAEA-S-2 (silver sulfide, *δ*^34^S_V-CDT_ = + 22.61‰), IAEA-S-3 (silver sulfide, *δ*^34^S_V-CDT_ = −32.49‰). Two in-house standards (BROC2. *δ*^34^S_V-CDT_ = + 11.55‰ ± 0.29‰, *n* = 8) and elemental microanalysis spirulina standard (B2162, *δ*^34^S_V-CDT_ = + 13.53 ± 0.17‰, *n* = 4) that are independently calibrated to the IAEA reference materials IAEA-S-1, IAEA-S-2 and IAEA-S-3, were used as a secondary check standards. All samples were analyzed in duplicate and gave an average 1 s reproducibility of ±0.3‰. Weight %S was calculated using an in-house broccoli standard broccoli (BROC2. S% = 0.84) calibrated using SOIL A (LECO – part number 502-309). Results are reported as per mil (‰) relative to V-CDT.

### Quantification and statistical analysis

#### Statistical analysis, data presentation and defining outliers

Outliers are expressed considering Tukey’s method for outlier detection. Sample distributions were visualised using density plot which are “smoothed” histogram of the sample distribution. Results were compared across the six middens using nonparametric pairwise comparisons between groups (Wilcoxon sum rank test,^83^) with the Benjamini, Hochberg, and Yekutiel corrections for multiple testing^84^ (Table S4). To compare the sample distributions, multiple tests were performed with two-sided alternative hypothesis. Given the presence of some statistical ties between the data, the probabilities obtained were not considered to be exact, and comparisons among groups are reported to be divided into three probability classes only: >0.05, ≤0.05, ≥0.01. Principal Component Analysis (PCA^85^) was used to reduce the dimensionality of the dataset and plot the variability of the data into bidimensional scatterplots (Figure S4). Since there was no statistically significant difference between domestic herbivores and wild herbivores for δ^13^C and δ^15^N, these two groups were merged into one class ’herbivores’. All analyses and graphics were made with the R software environment for statistical computing and graphics (ver. 4.4.2, R Core Team 2024).^86^

#### Isoscape development and provenencing

This approach relies solely on the ^87^Sr/^86^Sr provenancing map, as it offers a well-established and reliable method for geographical origin determination. In contrast, δ^34^S mapping, though promising, is still in its infancy and lacks the robust baseline data required for precise provenance analyses at this time (see supplementary text). ^87^Sr/^86^Sr outliers in Figure 5 are considered based on Tukey’s criteria (1.5x IQR) and outliers outside the estimated local range. Estimated local ranges are: ACC – 0.7075–0.7095; ECH – 0.7075–0.7095; SSB – 0.7075–0.7095; PTN – 0.708–0.7105; RMD – 0.7085–0.7125; WFD – 0.708–0.7115. A UK isoscape (Figure 4) was built using a random forest model, following the workflow of Bataille et al.^59^ Whilst the resolution of machine learning generated isoscapes provide reduced resolution compared to primary plant sampling they provide an effective means to map large areas, especially when incorporating purpose-specific sampling.^87^ Data used for training the model are from the current study and from^48^ (contains OS data © Crown Copyright and database right 2020 © and British Geological Survey materials © UKRI 2023; Figure S5). All the raster from Bataille et al.^59^ were used as external predictors in the model. A 10-fold cross validation was performed to assess the power of prediction, resulting in a Root Mean Squared Error (RMSE) of 0.0017 and an R^2^ of 0.52. A spatial uncertainty map was developed using a quantile random forest regression (*raster* R package;^88^), then halving the q_0.84_-q_0.16_ difference (i.e., lower and upper limit of a ∼68% interval).

Geographic probabilistic assignment of fauna (see Figure 5) was performed using the R package *assignR*^89^ and the isoscape map produced in this study. The isotope ratio of each sample was compared probabilistically to the isoscape and its associated prediction error, by using a Bayesian inversion method.^90^ The *a priori* assumption is that the sample can come equally from each cell of the isoscape. The posterior probability of sample origin is calculated at each grid cell, returning a raster containing a probability density surface per sample with its likely provenance (see e.g., Armaroli et al.^91^). Only the top 5% probability of origins were reported (Figure 5). Provenance groups used in the text are loosely defined through comparison of the *assignR* output maps.

## Supplemental information

Document S1. Figure S1–S5, Tables S1, S3, S4, and Methods S1–S3

Table S2. Full details of the 87Sr/86Sr and δ18O combined with previously published δ34S, δ15N, and δ13C 8
