# Supplementary material for: Diverse feasting networks at the end of the Bronze Age in Britain (c. 900-500 BCE) evidenced by multi-isotope analysis
Source: iScience. 2025 Sep 10;28(9):113271. doi: 10.1016/j.isci.2025.113271 (PMC12496200; doi:10.1016/j.isci.2025.113271)
Supplement: Document S1. Figure S1–S5, Tables S1, S3, S4, and Methods S1–S3 [file mmc1.pdf]

iScience, Volume *28*

Supplemental information

Diverse feasting networks at the end of the Bronze Age in Britain (c. 900-500 BCE) evidenced by multi-isotope analysis

Carmen Esposito, Angela L. Lamb, Morten B. Andersen, Marc-Alban Millet, Edward Inglis, Federico Lugli, Alexandra J. Nederbragt, and Richard Madgwick

Supplementary Text

Methods S1: $^{87}Sr/^{86}Sr$ taxon-specific patterns

Certain taxon-specific patterns are noteworthy (see Supplementary Table S3). All Cannings Cross caprine $^{87}Sr/^{86}Sr$ values range from 0.7077 to 0.7114, markedly broader than other taxa (although they account for the largest number of samples; n = 10). Pigs (n = 5) have a narrower range (0.7079-0.7093), in line with values from the single cattle, cervid and dog samples from the site. Only one outlier, a caprine, is identified (Figure 2A; ACC01 = 0.7114), but the Q–Q plot (Supplementary Figure S2) graphically highlights that ACC03 (0.7077) is also distinct from the normal distribution.

East Chisenbury $^{87}Sr/^{86}Sr$ values for caprines (n = 20) range from 0.7078 to 0.7110, again the widest of the different taxa. Pigs (n = 9) range between 0.7078 and 0.7096, the cattle (n = 6) range is 0.7080-0.7091, the deer (n = 2) range is 0.7080-0.7081, whilst the two dogs show a wide range of 0.7080-0.7099. All taxa show a narrow SD, with the exception of dogs (SD = 0.0013). Considering the distribution of all data, the Q–Q plot (Supplementary Figure S2) graphically highlights at least five outliers (2 caprine, 2 pigs, 1 dog), though only one outlier (ECH12 = 0.7110; Figure 2A).

Stanton St Bernard caprines (n = 15) and pigs (n = 15) have a broad range (0.7079-0.7109; 0.7079-0.7100), whereas cattle (n = 6, 0.7078-0.7088) and dogs (n = 2; 0.7080-0.7086) are more restricted. Means and medians are similar for all species with relatively small SDs. One caprine is an outlier (SSB05 = 0.7109; Figure 2A), but the Q–Q plot (Supplementary Figure S2), shows four possible outliers (1 caprine, 1 pig, 2 cattle).

In contrast to the other Wiltshire sites, Potterne caprine (n = 12) values have a narrower range (0.7081-0.7091, median and mean: 0.7085). Pig (n = 29) and cattle (n = 20) samples show a substantially broader range (0.7083-0.7147 and 0.7084-0.7105 respectively), albeit on larger datasets than the other sites, with pigs having a markedly larger SD (0.0016, compared to 0.0005 for cattle). Dogs at Potterne (n = 10) represent the only substantial canid dataset in the study and show a broad range (0.7085-0.7130) with a small IQR (0.0002) and broad SD (0.0013). Potterne shows 13 outliers (Figure 2A): 1 caprine, 9 pigs, 2 cattle and 1 dog, with 14 potential outliers evident on the Q-Q plot (9 pigs, 4 cattle and 1 dog, Supplementary Figure S2).

Caprines (n = 14), pigs (n = 25) and cattle (n = 11) have a broad range at Runnymede (0.7077-0.7122, 0.7089-0.7130, 0.7086-0.7138, respectively). The means are very close for those species, with pigs and cattle showing more radiogenic values. The only deer (RMD15 = 0.7098) and two dogs (RMD06 = 0.7097; RMD54 = 0.7102) show values consistent with the core of other data. Just one cattle sample is an outlier (Figure 2A; RMD51 = 0.7138), but the Q–Q plot (Supplementary Figure S2) shows 12 individuals that diverge from the normal distribution (3 caprines, 4 pigs and 5 cattle). Wallingford pigs (n = 10, 0.7089-0.7120) have a broader range compared to caprines (n = 15; 0.7079-0.7104) and cattle (n = 8; 7082-0.7101). Medians and means and SDs are very similar for all species. Only one pig is an outlier (WFD11 0.7120; Figure 2A), supported by the Q–Q plot (Supplementary Figure S2).

Methods S2: $^{87}Sr/^{86}Sr$ and $\delta^{34}S$ mapping limitations

$^{87}Sr/^{86}Sr$ and $\delta^{34}S$ data are often compared to local baselines or isoscapes (maps of bioavailable $^{87}Sr/^{86}Sr$) to distinguish local and non-local individuals. However, limitations in current methods continue to emerge. Primary bioavailable sampling is rarely exhaustive and usually limited to the closest area to the site (5 km ca.), leading to many more outliers than are really present. Constructing a thorough map of bioavailability is very expensive and time-consuming, and often impractical. Published biosphere maps are very useful starting points but frequently suffer from a limited sample density. Additionally, defining what is meant by local and non-local depends strongly on the different time periods and regions analysed. Finally, issues concerning equifinality of strontium isotope interpretations are well known in archaeological studies [1]. Different geological areas could have similar $^{87}Sr/^{86}Sr$ values, often making it impossible to distinguish outliers using $^{87}Sr/^{86}Sr$ data alone.

The more refined UK sulfur isotope biosphere map presented by Evans et al. [2] represents a major step forward compared to previous iterations, which principally presented a contrast between coastal and inland areas. It is now clear that wetlands tend to produce negative values [3], and estuaries can generate marine-affected values inland [2]. However, issues of modern pollution, though markedly reduced [3], persist in lowering biosphere values compared to archaeological time periods, meaning modern plants cannot always be relied on as a good analogue for archaeological periods (see [4]). In addition, the nature of $\delta^{34}S$ cycling and its impact on bioavailability over time is poorly understood providing further uncertainty in relation to the application of modern isoscapes. Figure 2B shows very good alignment between the primary plant analyses and the Evans et al. [2] map, with primary samples either mirroring the map or providing a more tightly defined local range within the BGS range which might be considered regional. However, the alignment with archaeological data is less consistent. It would be expected that the majority of animals are consistent with a local or at least regional origin (as supported by $^{87}Sr/^{86}Sr$ data), but for some sites, the local range falls on the periphery of the faunal range. This is clearest in the chalkland sites of East Chisenbury, All Cannings Cross and Stanton St. Bernard and, to a lesser extent, at Runnymede. This raises questions about the efficacy of modern plant analysis for $\delta^{34}S$ biosphere mapping, in a densely populated area like Britain at least. With all plant ranges being lower, it is plausible that modern emissions have impacted results, an issue that has markedly reduced in Britain since the Clean Air Act 1956, but remains a problem (see [5]). However, this pattern is inconsistent and Wallingford and Potterne plants align closely to faunal data. Therefore, it does not appear that there is a consistent offset between modern plants and faunal samples, but this varies regionally. Therefore, the modern plant $\delta^{34}S$ data and the Evans et al. [2] map are used cautiously and natural breaks in the data and outlying samples are also considered in identifying non-local individuals. This demonstrates the complexity of $\delta^{34}S$ mapping and indicates that there is no one-size-fits-all approach.

Some limitations are also observable with $^{87}Sr/^{86}Sr$ baselines, with substantial numbers of animals having values outwith the local range defined by primary plant analysis and the published biosphere map of Britain [2]. Generally, outliers highlighted in the results section broadly align with those that are outwith the biosphere range. However, all sites except Potterne (which has 13 outliers) and Wallingford show a greater number of non-locals than the statistically defined outliers (despite conservative regional ranges being defined). If the primary plant samples are considered separately,

then all midden sites have a majority of non-local animals. This likely indicates that the primary sampling was not extensive enough to characterise bioavailability in the grazing land surrounding the sites and therefore some locally-raised animals are outside the local range. This is to be expected given the limits of the plant sampling strategy – it is highly likely that some animals grazed or acquired food beyond the immediate vicinity of the site even if they were locally raised (e.g. seasonal pasturage exploitation of woodland resources for pigs).

Methods S3: $\delta^{18}O$ mapping limitations

As stated in the results section, variation in $\delta^{18}O$ in water sources across the landscape cannot easily be mapped in relation to the expected values in pig enamel. Therefore, results need to be interpreted in the context of a substantial dataset. In our case, middens were interpreted through inter-site comparison in the context of a substantial dataset of Late Neolithic pigs from southern Britain, which were analysed using the same protocol. This dataset is not used for the identification of non-locals but rather in the exploration of data variation and outliers. Both similarities and differences are observable in comparing these data to the Neolithic sample, but variable sample sizes make direct comparisons challenging. It is worth noting that there is strong multi-isotope evidence for wide-ranging origins in the Neolithic pigs [6]. Overall, the Late Neolithic pigs from Wiltshire and Dorset show lower values than the LBA-EIA middens from Wiltshire, with means being lower for all four of the later sites, despite being in similar regional locations. The Thames Valley middens, however, present similar ranges to the Neolithic sites (all having means between 25.0‰ and 25.9‰) in spite of being more easterly meaning they would be expected to have lower $\delta^{18}O$ values. Diet and husbandry may be responsible for this. $\delta^{15}N$ isotope values are more wide-ranging in the smaller LBA-EIA sample. Pigs are clearly more omnivorous in these later phases of prehistory (see [7]) and the wider range of dietary resources, likely including a substantial quantity of human food waste, could well impact $\delta^{18}O$ values causing a greater range. It is noteworthy that a single outlier in the Neolithic dataset had very high $\delta^{18}O$ and $\delta^{15}N$ (MP107, 28.9‰, 9.1‰ respectively). Therefore, it is possible that a greater reliance on $\delta^{18}O$ enriched meal waste (e.g. stewed food, slops and waste from cheese making) means that the LBA-EIA sample has higher $\delta^{18}O$ and $\delta^{15}N$ than the Neolithic sample. Consequently, it is likely that dietary variation means $\delta^{18}O$ is of limited use in defining origins but is of value in exploring the range of origins and inter-site differences. Differences in the range of water sources used by pigs provide an alternative explanation for the variation. Overall, the $\delta^{18}O$ isotope data support the patterns of mobility and catchment in the other isotope proxies.

Supplementary Figures

Fig. S1. Box plots showing isotope variation in modern plant samples collected from different midden sites. (A) δ^{34}S and (B) ^{87}Sr/^{86}Sr ratios with mean +/- 1 standard deviation (SD) of plants. Mean and +/- 1 SD is represented through crossbars. Midden sites: ACC = All Cannings Cross; ECH = East Chisenbury; PTN = Potterne; RMD = Runnymede; SSB = Stanton St Bernard; WFD = Wallingford (BGS©UKRI).

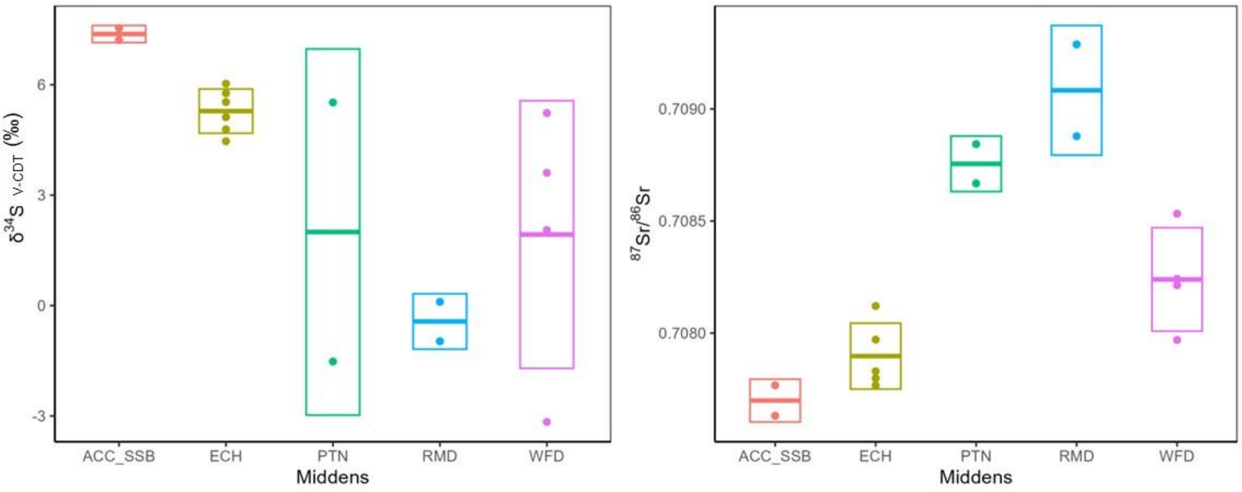

Fig. S2. Normal probability plot or Quantile–Quantile plot (A) and kernel density plot (B) of $^{87}Sr/^{86}Sr$ faunal values of different midden sites. The Q–Q plot compares the expected normal (black line) versus data quantiles. A density plot visualizes data distribution over a continuous interval – the peaks of a density plot display where values are concentrated.

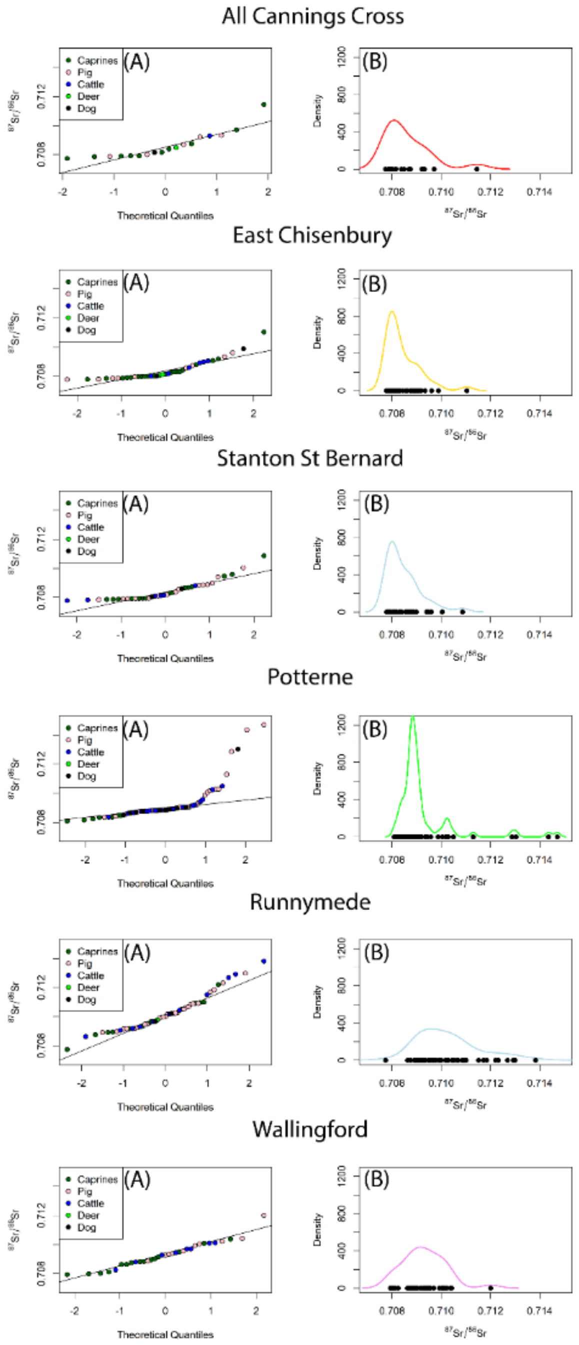

Fig. S3. Box and whisker plots of $^{87}Sr/^{86}Sr$ for midden sites divided by species. Midden sites: Midden sites: ACC = All Cannings Cross; ECH = East Chisenbury; PTN = Potterne; RMD = Runnymede; SSB = Stanton St Bernard; WFD = Wallingford (BGS©UKRI).

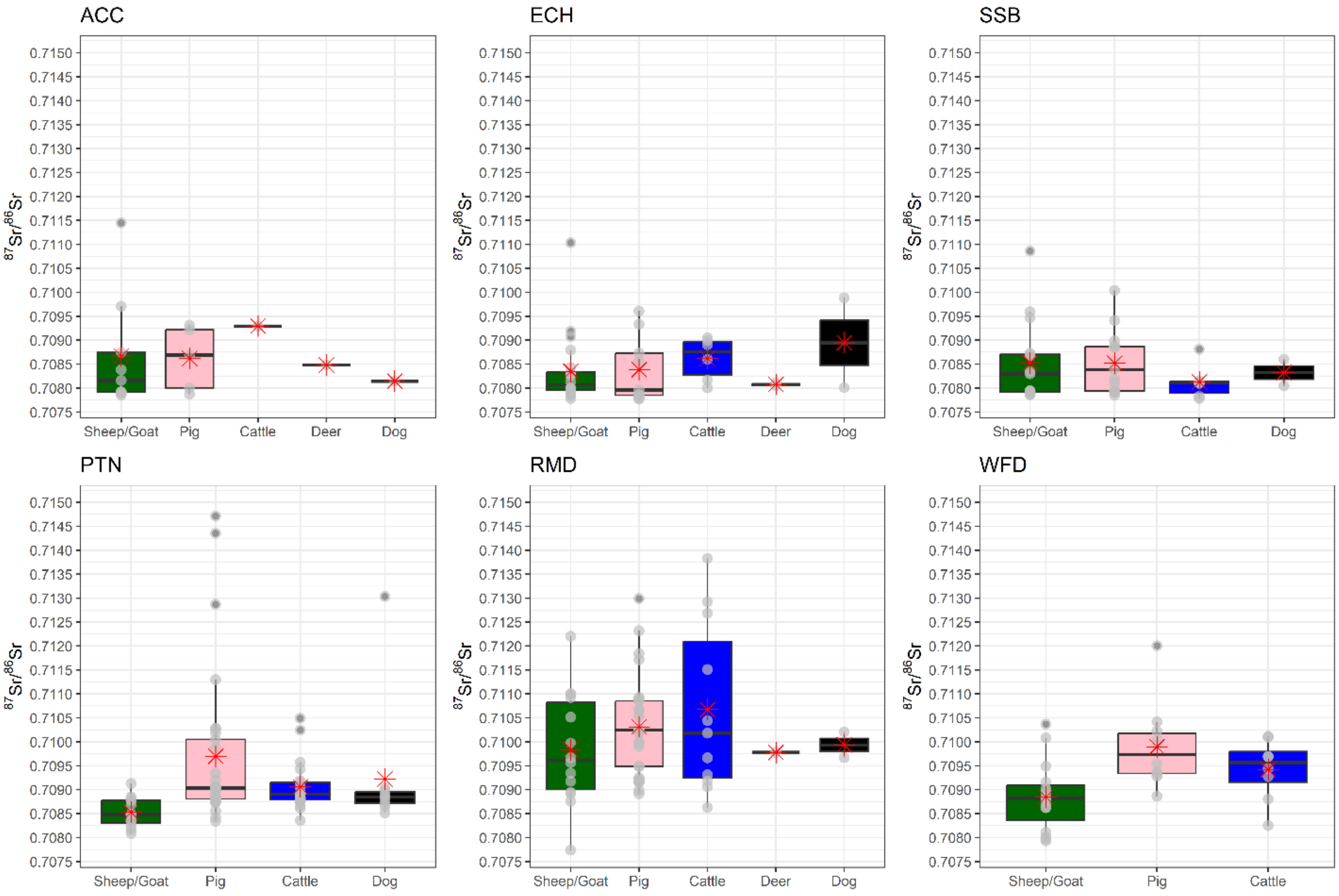

Fig. S4. Convex hull plot of the PCA individual scores on the two first principal components based on $\delta^{13}C$, $\delta^{15}N$, $\delta^{34}S$, $^{87}Sr/^{86}Sr$, divided by middens. Midden sites: ACC = All Cannings Cross; ECH = East Chisenbury; PTN = Potterne; RMD = Runnymede; SSB = Stanton St Bernard; WFD = Wallingford (BGS©UKRI).

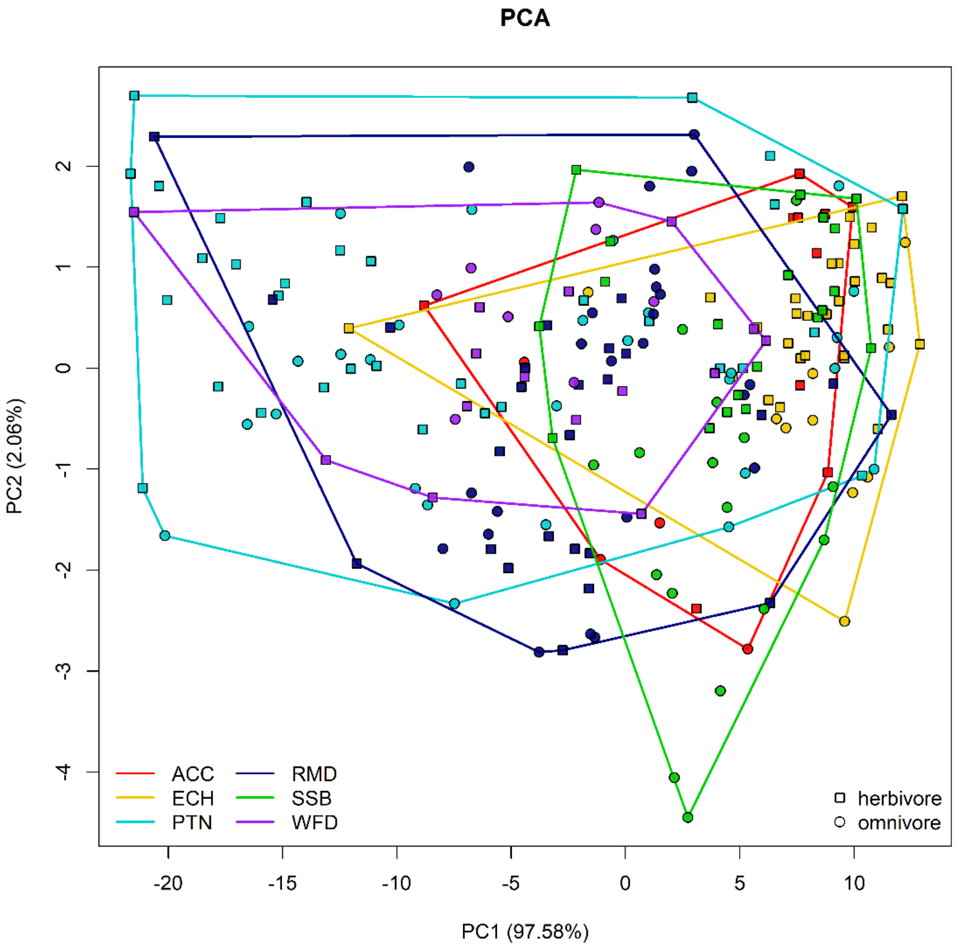

Fig. S5. $^{87}Sr/^{86}Sr$ data from the UK. Data used for building the isoscape, including plant samples from this work and BGS bioavailable data. Data are coloured with a quantile scale (min, $q_{0.1}$, median, $q_{0.9}$, max), based on their Sr isotope ratio. Contains OS data © Crown Copyright and database right 2020 and British Geological Survey materials © UKRI 2023.

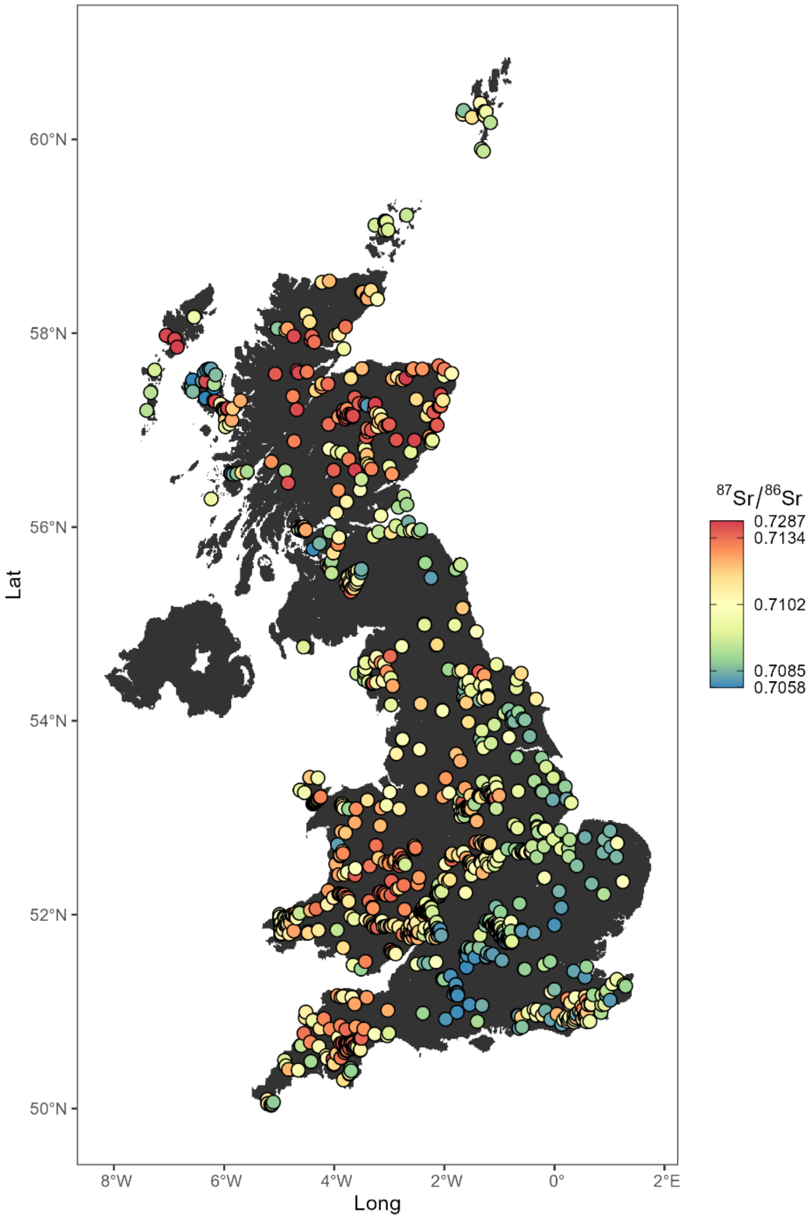

Supplementary Tables

Table S1. Plants (shrubs and hedges) $^{87}Sr/^{86}Sr$ and $\delta^{34}S$ values for the different midden sites. Midden sites: ACC = All Cannings Cross; ECH = East Chisenbury; PTN = Potterne; RMD = Runnymede; SSB = Stanton St Bernard; WFD = Wallingford.

Sample	Latitude	Longitude	$^{87}Sr/^{86}Sr$	2se	$\delta^{34}S_{V\text{-}CDT}$	%S	Reps
ACC/SSBP01	51.369167	-1.889444	0.7076	1.93E-05	7.2	0.28	1
ACC/SSBP02	51.368994	-1.886215	0.7078	3.28E-05	7.5	0.21	1
ECHPA	51.277642	-1.791813	0.7078	7.00E-06	6.0	0.08	1
ECHPB	51.277747	-1.792365	0.7080	1.00E-05	5.1	0.16	1
ECHPC	51.277539	-1.791914	-	-	4.5	0.15	1
ECHPD	51.277605	-1.792146	0.7081	6.00E-06	4.8	0.18	1
ECHPE	51.277642	-1.791813	0.7078	8.00E-06	5.5	0.08	1
ECHPF	51.277642	-1.791813	0.7078	7.00E-06	5.8	0.19	1
PTNP01	51.336583	-2.033194	0.7087	2.70E-05	-1.5	0.37	1
PTNP02	51.330642	-2.007671	0.7088	3.00E-05	5.5	0.26	1
RMDP01	51.437477	-0.548501	0.7093	1.75E-05	0.1	0.30	2
RMDP02	51.436798	-0.538024	0.7089	1.70E-05	-1.0	0.42	1
WFDP01	51.590989	-1.120638	0.7080	1.97E-05	2.1	0.28	**1**
WFDP02	51.335216	-2.026375	0.7085	1.65E-05	-3.2	0.36	1
WFDP03	51.583611	-1.156139	0.7082	2.90E-05	5.2	0.47	1
WFDP04	51.605651	-1.217208	0.7082	1.94E-05	3.6	0.29	1

Table S3. Summary statistics for $^{87}Sr/^{86}Sr$ and $\delta^{18}O$ for different species at midden sites. Midden sites: ACC = All Cannings Cross; ECH = East Chisenbury; PTN = Potterne; RMD = Runnymede; SSB = Stanton St Bernard; WFD = Wallingford.

		$^{87}Sr/^{86}Sr$					$\delta^{18}O_{V\text{-}SMOW}$
		Sheep/Goat	Pig	Cattle	Cervidae	Dog	Pig
ACC	n	10	5	1	1	1	5
	median	0.7080	0.7087	-	-	-	27.1
	mean	0.7086	0.7086	-	-	-	26.6
	min	0.7077	0.7079	0.7093	0.7085	0.7081	25.3
	max	0.7114	0.7093	0.7093	0.7085	0.7081	27.7
	IQR	0.0007	0.0012	-	-	-	2.0
	1 SD	0.0012	0.0007	-	-	-	1.1
ECH	n	20	9	6	2	2	9
	median	0.7081	0.7080	0.7088	0.7081	0.7089	25.5
	mean	0.7084	0.7084	0.7086	0.7081	0.7089	26.5
	min	0.7078	0.7078	0.7080	0.7080	0.7080	24.6
	max	0.7110	0.7096	0.7091	0.7081	0.7099	28.8
	IQR	0.0004	0.0009	0.0007	0.0007	0.0009	3.2
	1 SD	0.0007	0.0007	0.0004	0.0000	0.0013	1.8
SSB	n	15	15	6	0	2	15
	median	0.7083	0.7084	0.7081	-	0.7083	27.2
	mean	0.7085	0.7085	0.7081	-	0.7083	26.7
	min	0.7079	0.7079	0.7078	-	0.7080	24.1
	max	0.7109	0.7100	0.7088	-	0.7086	28.5
	IQR	0.0008	0.0009	0.0002	-	0.0003	1.5
	1 SD	0.0009	0.0006	0.0004	-	0.0004	1.4
PTN	n	12	29	20	0	10	29
	median	0.7085	0.7090	0.7089	-	0.7088	26.2
	mean	0.7085	0.7097	0.7091	-	0.7092	26.4
	min	0.7081	0.7083	0.7084	-	0.7085	24.4
	max	0.7091	0.7147	0.7105	-	0.7130	29.2
	IQR	0.0005	0.0012	0.0004	-	0.0002	2.1
	1 SD	0.0003	0.0016	0.0005	-	0.0013	1.4
RMD	n	14	25	11	1	2	25
	median	0.7096	0.7102	0.7102	-	0.7099	25.2
	mean	0.7098	0.7103	0.7107	-	0.7099	25.5
	min	0.7077	0.7089	0.7086	0.7098	0.7097	23.5
	max	0.7122	0.7130	0.7138	0.7098	0.7102	28.1
	IQR	0.0018	0.0014	0.0029	-	0.0003	1.8
	1 SD	0.0012	0.0011	0.0018	-	0.0004	1.2
WFD	n	15	10	8	0	0	10
	median	0.7088	0.7097	0.7096	-	-	25.2
	mean	0.7089	0.7099	0.7094	-	-	25.3
	min	0.7079	0.7089	0.7082	-	-	23.2
	max	0.7104	0.7120	0.7101	-	-	27.4
	IQR	0.0007	0.0008	0.0006	-	-	1.2
	1 SD	0.0007	0.0009	0.0006	-	-	1.2

Table S4. Matrix of paired Wilcoxon sum rank test significance (two tailed alternative hypothesis calculation of probabilities). Lower left matrix = $^{87}Sr/^{86}Sr$. Upper right matrix = $\delta^{34}S$. ns= $p>0.05$; *= $p\leq0.05$; **=$p\leq0.01$. Midden sites: ACC = All Cannings Cross; ECH = East Chisenbury; PTN = Potterne; RMD = Runnymede; SSB = Stanton St Bernard; WFD = Wallingford. Notations in parentheses indicate the direction of significance (using single tailed alternative hypothesis calculation of probabilities), with < indicating significantly higher values in the site listed in the column and > indicating significant higher values in the site listed on the row.

$\delta^{34}S$ (upper right) / $^{87}Sr/^{86}Sr$ (lower left)	ACC	ECH	PTN	RMD	SSB	WFD
ACC	-	*(<)	**(>)	**(>)	ns	**(>)
ECH	ns	-	**(>)	**(>)	**(>)	**(>)
PTN	**(>)	**(>)	-	*	**(>)	ns
RMD	**(>)	**(>)	**(>)	-	**	ns
SSB	ns	ns	**(<)	**(<)	-	**(>)
WFD	**(>)	**(>)	ns	**(<)	**(>)	-

Supplementary Reference

1. Holt, E., Evans, J.A., and Madgwick, R. (2021). Strontium (87Sr/86Sr) mapping: A critical review of methods and approaches. Earth-Science Reviews *216*, 103593. https://doi.org/10.1016/j.earscirev.2021.103593.
2. Evans, J.A., Chenery, C.A., Mee, K., and Marchant, A.P. (2022). Biosphere isotope domains GB (V2): Interactive website. British Geological Survey (Interactive Resource). doi: 10.5285/2ce/7fc22-1b6e-4679-968f-42058c0120fb.
3. Lamb, A.L., Chenery, C.A., Madgwick, R., and Evans, J.A. (2023). Wet feet: Developing sulfur isotope provenance methods to identify wetland inhabitants. Royal Society Open Science *10*, 230391. 10.1098/rsos.230391.
4. Tarrant, D., and Richards, M.P. (2024). Modern plants and sulfur isoscapes - A review, discussion, and construction of a pilot δ34S isoscape for mobility and provenance studies. Rapid Communications in Mass Spectrometry *38*, e9908. 10.1002/rcm.9908.
5. Zhao, F.J., Spiro, B., Poulton, P.R., and McGrath, S.P. (1998). Use of sulfur isotope ratios to determine anthropogenic sulfur signals in a grassland ecosystem. Environmental Science & Technology *32*, 2288-2291. 10.1021/es980157f.
6. Madgwick, R., Lamb, A.L., Sloane, H., Nederbragt, A.J., Albarella, U., Pearson, M.P., and Evans, J.A. (2019). Multi-isotope analysis reveals that feasts in the Stonehenge environs and across Wessex drew people and animals from throughout Britain. Science Advances *5*, eaau6078. 10.1126/sciadv.aau6078.
7. Madgwick, R., Mulville, J., and Stevens, R.E. (2012). Diversity in foddering strategy and herd management in Late Bronze Age Britain: An isotopic investigation of pigs and other fauna from two midden sites. Environmental Archaeology *17*, 126-140. 10.1179/1461410312Z.00000000011.
8. Madgwick, R., Esposito, C., and Lamb, A.L. (2023). Farming and feasting during the Bronze Age–Iron Age transition in Britain (ca. 900–500 bce): Multi-isotope evidence for societal change. Frontiers in Environmental Archaeology *2:122158*.
